# Synergistic *sp*-C/*sp*-N Anchoring of Metal Single Atoms on Graphdiyne for Enhanced Microwave Absorption

**DOI:** 10.1007/s40820-026-02187-8

**Published:** 2026-04-28

**Authors:** Yihao Fan, Haichuan Cheng, Pengyu Deng, Jianfeng Wu, Baoliang Zhang

**Affiliations:** 1 School of Chemistry and Chemical Engineering, Northwestern Polytechnical University, Xi’an, 710072 People’s Republic of China; 2 Xi’an Key Laboratory of Functional Organic Porous Materials, Northwestern Polytechnical University, Xi’an, 710129 People’s Republic of China; 3 Shaanxi Engineering and Research Center for Functional Polymers On Adsorption and Separation, Sunresins New Materials Co. Ltd., Xi’an, 710072 People’s Republic of China

**Keywords:** Graphdiyne, Microwave absorption, Single atoms, FeN_2_C_2_, Nanosphere

## Abstract

Enhancing the conduction and polarization properties of the emerging two-dimensional carbon material graphdiyne (GDY) represents a crucial step in broadening its application in microwave absorption. A novel strategy was proposed to improve the microwave absorption performance of GDY through precise regulation of single-atom structures. Using three-dimensional spherical GDY as a substrate, Two Fe single-atom absorbers were successfully constructed: one anchored by Fe–N-GDY (FeN_2_C_2_) via *sp*-N/*sp*-C cooperative coordination, and the other anchored by Fe-GDY (FeC_4_) via *sp*-C coordination alone. Combined experimental characterization and theoretical calculations revealed that the FeN_2_C_2_ configuration induces stronger charge transfer and dipole polarization. This effect synergistically optimizes both the dielectric loss and impedance matching of the material. Consequently, the optimal sample Fe–N-GDY achieved an effective absorption bandwidth of 5.98 GHz at a matched thickness of 2.0 mm, with a minimum reflection loss of −51.2 dB. The strategy was further extended to multiple 3*d* transition metals (Cr, Mn, Co, Ni, Cu, and Zn). Results indicate that Group VIII elements (Fe, Co, Ni) exhibit superior performance in practical materials due to their electronic structures that favor balancing polarization and conduction losses. Radar cross section simulations confirm the exceptional attenuation capabilities of this series of absorbers in real-world scenarios. This work not only pioneers new applications for GDY in microwave absorption but also establishes a theoretical foundation for rationally designing atomically precise electromagnetic functional materials by revealing the “single-atom structure–property” correlation.

**Supplementary Information:**

The online version contains supplementary material available at 10.1007/s40820-026-02187-8.

## Highlights

- The metal single-atom doping strategy was systematically applied to graphdiyne (GDY), an emerging two-dimensional carbon material, and a series of M-N-GDY single-atom microwave absorbers were successfully fabricated for the first time.
- Combining X-ray absorption fine structure and density functional theory calculations, the influence of the central atom coordination structure (FeN_2_C_2_, FeC_4_) on the overall electronic properties was elucidated.
- The optimal sample Fe-N-GDY achieved an effective absorption bandwidth of 5.98 GHz at a matched thickness of 2.0 mm, with a minimum reflection loss of -51.2 dB.

**Supplementary Information:**

The online version contains supplementary material available at 10.1007/s40820-026-02187-8.

## Graphical abstract

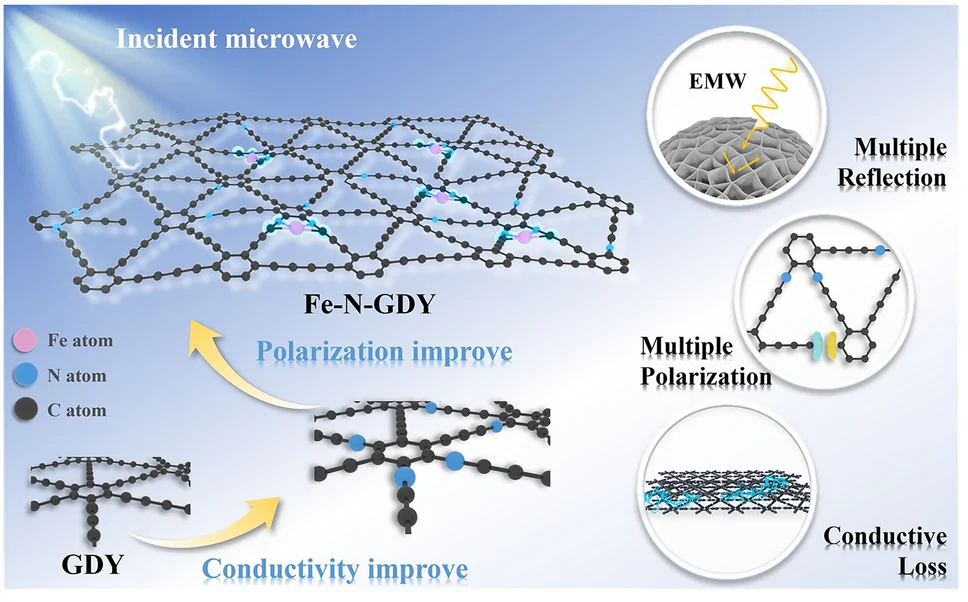

## Introduction

With the rapid iteration and upgrade of electromagnetic wave application technologies [1], demands such as enhancing the stealth performance of military equipment, optimizing the electromagnetic compatibility of high-density electronic systems [2], and protecting against high-frequency electromagnetic pollution have become increasingly urgent [3, 4]. These collectively constitute multiple technical challenges in the field of current electromagnetic functional materials [5, 6]. Against this background, how to design and fabricate new microwave absorbing materials (MAMs) featuring lightweight, broad absorption bandwidth, and high loss efficiency [7, 8] based on the "integrated structure–function" design concept has become one of the hot and challenging topics in current research [9]. Among various MAMs, carbon-based MAMs [10–12], such as graphene [13], carbon nanotubes [14], and carbon fibers [15], have emerged as one of the key materials for addressing the needs of electromagnetic pollution control and countering complex high-precision detection [16–18], owing to their advantages of low density, excellent chemical inertness [19], outstanding microwave attenuation capability [20], and high designability [21–23].

Graphdiyne (GDY), an emerging two-dimensional carbon allotrope [24], is composed of *sp*- and *sp*^2^-hybridized carbon atoms. The unique π-conjugated structure [25] endows GDY with numerous excellent properties [26]. These include abundant nanochannels, high specific surface area, high carrier mobility, as well as considerable mechanical strength and thermal stability [27]. These properties align well with the key requirements for microwave absorbing materials: lightweight, strong loss, and structural stability [28]. Previous research by this group [29] has confirmed that appropriate thermal treatment can effectively remove functional groups and oligomers from the GDY surface, thereby activating its dielectric loss properties. Nonetheless, the microwave absorption performance of pristine GDY material still has an upper limit. The tunable ranges of its complex permittivity are relatively fixed, and its loss capability is limited, making it difficult to achieve ideal microwave absorption across a broad frequency range. Therefore, how to effectively enhance its electromagnetic wave loss capability becomes crucial.

The strategy of constructing three-dimensional transition metal single atoms on carbon-based substrates [30–32] has become a frontier approach in microwave absorbing material research [33, 34]. This strategy retains the lightweight characteristics of the carbon material while utilizing the charge transfer between atomically dispersed metal atoms and the substrate [35], which can significantly enhance the material's interfacial polarization and dipole polarization loss capabilities [36]. However, the stable anchoring of single atoms strongly depends on the stable coordination environment provided by the substrate. Existing research has predominantly focused on using graphene as the substrate [37, 38], which requires additional defect sites and chemical doping [39], and faces issues of uneven dispersion and low loading capacity. In comparison, GDY offers inherent advantages for anchoring single atoms due to its intrinsic *sp/sp*^2^-hybridized carbon network [40]. The *sp*-hybridized carbon atoms in its alkyne-rich structure can act as strong Lewis base sites, forming strong chemical bonds with metal atoms, thereby enabling higher loading capacity and more uniform dispersion of single atoms [41]. More importantly, through substitutional doping with nitrogen atoms, a composite coordination structure containing both *sp*-N and *sp*-C hybridized atoms can be constructed on the GDY framework [42]. Studies have shown that such *sp*-N hybridization can induce additional electronic states near the Fermi level, which can further strengthen the electronic coupling and charge transfer efficiency between the single-atom metal center and the GDY substrate [43]. For single-atom loaded absorbers, changes in the single atom's coordination environment can cause a shift in its *d*-band center [42], thereby profoundly influencing the overall conductivity and polarization relaxation behavior of the absorber [44]. However, how to construct the optimal coordination structure on GDY through precise atomic-level doping, elucidate the impact of changes in single-atom coordination structure on the electronic properties of GDY, and clarify its mode of action in altering conduction and polarization, remains to be thoroughly investigated.

Based on this, using spherical GDY prepared by emulsion interfacial polymerization as the substrate, Fe single atoms were anchored via *sp*-N/*sp*-C co-coordination (FeN_2_C_2_) and solely via *sp*-C coordination (FeC_4_), respectively. Two single-atom-loaded adsorbents, Fe–N-GDY and Fe-GDY, were successfully synthesized. Subsequently, systematic material characterization, microwave absorption performance testing, and density functional theory (DFT) calculations were combined to deeply reveal the regulatory principles and underlying mechanisms of Fe single-atom coordination structures on the electromagnetic parameters of GDY. Performance evaluation indicated that the introduction of the FeN_2_C_2_ coordination structure provided the most significant performance enhancement. The resulting absorber, Fe–N-GDY, achieved an effective absorption bandwidth (EAB) of 5.98 GHz at a matching thickness of 2.0 mm, demonstrating exceptional broadband microwave absorption capability. Furthermore, the research system was extended to a series of 3*d* transition metals, preparing M–N-GDY (M = Cr, Mn, Fe, Co, Ni, Cu, Zn) with similar MC_2_N_2_ coordination configurations. Among them, the M–N-GDY loaded with Group VIII elements (Fe, Co, Ni) exhibited more superior microwave absorption performance. This work innovatively introduces metal single-atom doping engineering into the GDY-based absorber material system. It not only provides new insights for expanding the functional applications of GDY but also offers crucial theoretical and experimental foundations for rationally designing atomically precise electromagnetic functional materials on demand in the future.

## Experimental Section

### Materials

Hexakis-[(trimethylsilyl) ethynyl] benzene (HEB-TMS, 97%), tetrabutylammonium fluoride (TBAF, 1 M in THF), and pyridine (99%) were purchased from Adamas-beta®. Dichloroethane (DCE, 99%), anhydrous ethanol, Cupric acetate monohydrate (99%) and Ammonia Water (25%–28%) were purchased from Guangdong Guanghua Technology Co., Ltd.. Hexadecyl trimethyl ammonium bromide (CTAB) (99%) was purchased from Shanghai Macklin Biochemical Technology Co., Ltd.. All chemicals were analytical grade (AR) without any further purification. The deionized water used was produced by the water purification system in the laboratory.

### Preparation of GDY

In a three-neck flask, 8.4 g CTAB was dissolved in 60 mL aqueous solution containing 2.4 g pyridine. The mixture was heated to 50 °C under continuous stirring. Separately, 80 mg HEB-TMS was dissolved in 30 mL DCE, followed by adding 30 μL of 1 M TBAF in tetrahydrofuran. This solution was added dropwise to the flask and stirred at 350 rpm for 5 min. The emulsion was sonicated (40 kHz, 300 W) for 20 min. Subsequently, 3 mL aqueous solution containing 25 mg copper (II) acetate was added. The reaction proceeded for 24 h in the dark. The resulting GDY particles were collected by centrifugation, washed with ethanol and aqueous ammonia solution, then dry under vacuum.

### Synthesis of Fe–N-GDY and Control Samples

First, 30 mg of GDY was ultrasonically dispersed in 30 mL of deionized water, and the resulting dispersion was subjected to magnetic stirring. Subsequently, 2 mL of an aqueous FeCl_3_ solution (C = 0.01 mol L^−1^) was added dropwise into the dispersion. After stirring for 3 h, the mixture was filtered and washed to remove unadsorbed Fe^3+^ ions. Then, the Fe^3+^-adsorbed GDY was mixed with 45 mg of melamine and redispersed in 30 mL of ethanol, followed by stirring for 1 h. The solvent was removed using a rotary evaporator. Finally, the resulting solid was annealed in a tube furnace under an Ar atmosphere to obtain the final powder, denoted as Fe–N-GDY.

For comparison, control samples were prepared under conditions consistent with those for Fe–N-GDY: Fe NPs/Fe–N-GDY was obtained by increasing the concentration of the added FeCl_3_ solution (C = 0.1 mol L^−1^); Fe-GDY was prepared in the absence of an N source; N-GDY was synthesized in the absence of an Fe source; GDY-900 was obtained by subjecting pristine GDY to the same annealing procedure.

### Synthesis of M–N-GDY

The M–N-GDY samples were prepared following the same procedure as that for Fe–N-GDY, with the only modification being the type of metal element in the aqueous metal chloride solution used.

### Characterizations

The morphology and internal structure of the samples were observed by field emission scanning electron microscope (SEM, Verios G4, FEI), Transmission Electron Microscope (TEM, FEI Talos F200X, FEI) and Double Cs Corrector Transmission Electron Microscope (Double Cs-corrected TEM, Themis Z, FEI). Raman spectra were obtained by micro confocal Raman spectroscopy (Invia Qontor, Renishaw) under 633 nm laser excitation. The specific surface area and pore size distribution of the samples were measured using a nitrogen adsorption meter (Tristar 3020, Micromeritics) using the BET method. The diffraction peaks of the samples were analyzed using X-ray diffraction (XRD, D8 Advance, Bruker) in the range of 10 degrees to 80 degrees. The heat treatment process was carried out in a tube furnace (KMTF-1100–30220, Kemi). X-ray photoelectron spectroscopy (XPS) measurements were operated by Kratos AXIS Supra spectrometer with Al Kα radiation as the excitation sources. Fe K-edge X-ray absorption fine structure analyses were performed with Si(111) crystal monochromators at the BL14W Beam line at the Shanghai Synchrotron Radiation Facility (SSRF) (Shanghai, China). The Fe content was determined by Inductively coupled plasma Mass Spectrometry (ICP-MS, Avoi220Max, PerkinElmer).

## Results and Discussion

### Synthesis and Characterization of Fe–N-GDY

**Fig. 1:**
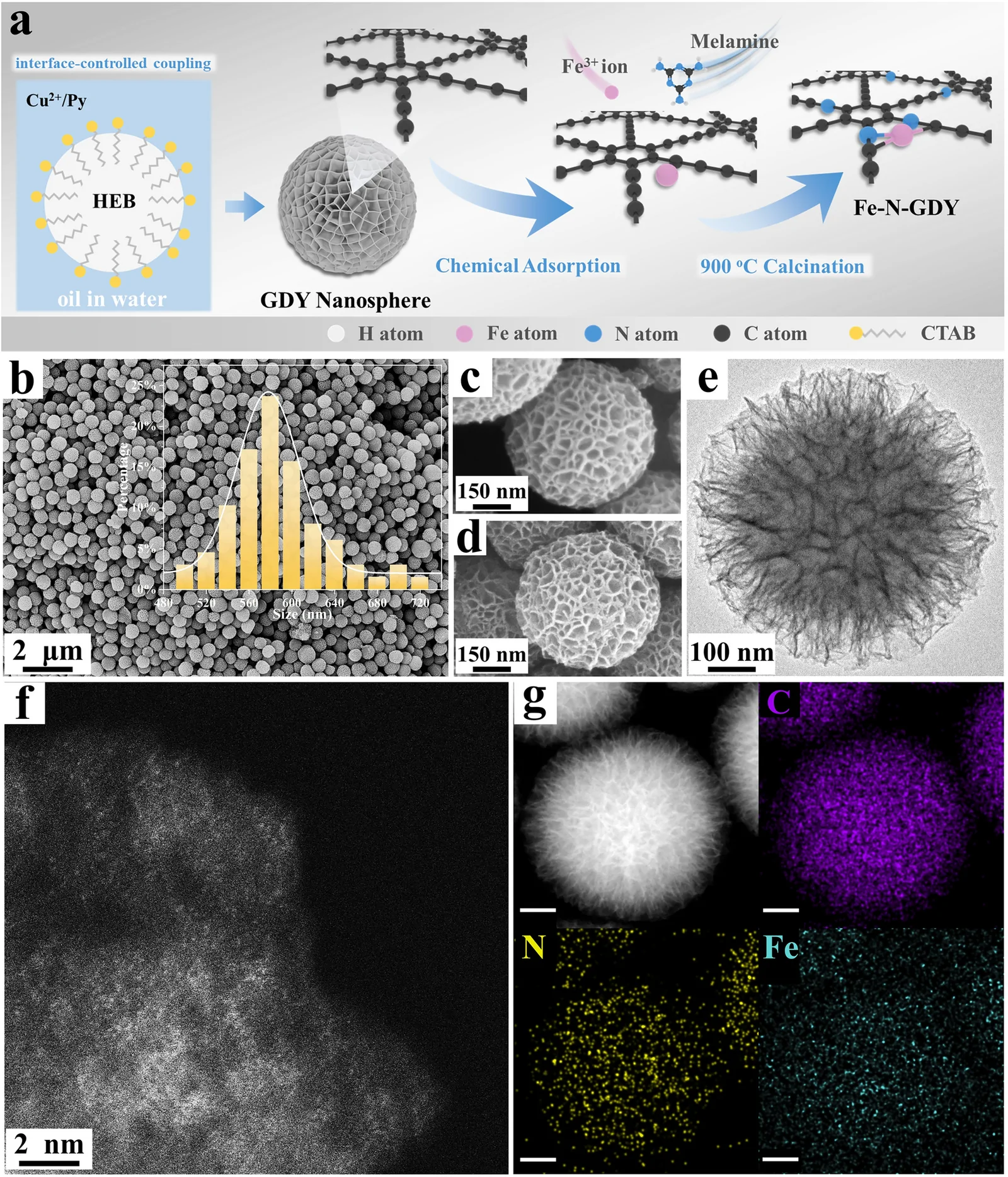
**a** Schematic illustration of the preparation of Fe–N-GDY. **b** SEM image of Fe–N-GDY particles. High-magnification SEM image of **c** GDY and **d** Fe–N-GDY. **e** TEM image, **f** double Cs-corrected TEM image, and **g** elemental mapping of Fe–N-GDY (scale bar = 100 nm)

Figure 1a schematically illustrates the synthesis route of Fe–N-GDY. First, spherical Cu_2_O/ GDY particles were synthesized via an oil-in-water (O/W) microemulsion system stabilized by CTAB. In this system, a DCE solution containing HEB-TMS and the deprotecting agent TBAF served as the dispersed phase, while an aqueous solution containing pyridine and the catalyst copper acetate acted as the continuous phase. Subsequently, the deprotonated hexaethynylbenzene (HEB) precursor formed a coordination intermediate with Cu^2+^ [45] and grew at the oil/water interface via intermolecular Glaser–Hay coupling reaction, gradually constructing the GDY network. As the HEB in the oil droplets was depleted, a spherical Cu_2_O/GDY precursor was ultimately formed, using the microemulsion droplets as a soft template. Afterward, the GDY particles, after ammonia water treatment to remove Cu_2_O clusters, were dispersed in an aqueous FeCl_3_ solution of specific concentration. Benefiting from the semi-open cavity structure of the GDY microspheres and the C18 nanochannels within the GDY framework, Fe^3+^ ions were efficiently and uniformly adsorbed onto the GDY substrate. Then, unadsorbed free Fe^3+^ was removed by centrifugation and washing, and the Fe^3+^-adsorbed GDY particles were thoroughly mixed with a specific stoichiometric ratio of melamine. Finally, the mixture was annealed under an Ar atmosphere. During this process, nitrogen-containing species generated from the pyrolysis of melamine interacted with the GDY framework, ultimately achieving the preparation of Fe single atoms (Fe–N-GDY) co-anchored by *sp*-N and *sp*-C hybridized atoms.

Figure 1b displays the SEM image of Fe–N-GDY particles. Due to the spatial confinement effect provided by the emulsion system [46], the obtained microspheres exhibit high sphericity and uniform size, with particle diameters concentrated around 580 nm, successfully inheriting the original morphology of the precursor GDY (Fig. S1). Figure 1c, d shows high-magnification SEM images of GDY and Fe–N-GDY, respectively, where a unique nanosheet structure grown on the surface of the microspheres can be observed. This three-dimensional porous layered structure not only provides abundant sites for anchoring metal single atoms, but its interwoven and interconnected internal channels are also expected to optimize the propagation path of electromagnetic waves, enhancing the attenuation of incident microwaves by inducing multiple reflections and scattering [47]. After the annealing treatment, the nanowalls became thinner, and the specific surface area increased from 228.5 m^2^ g^−1^ before annealing to 794.62 m^2^ g^−1^ (Fig. S2a), representing an increase in approximately 248%. Analysis of their pore size distribution curves (Fig. S2b) shows that both samples exhibit a sharp peak at 4.5 nm. According to the infrared spectroscopy analysis (Fig. S3), it can be concluded that the annealing treatment effectively removed residual CTAB templating agents and incompletely cross-linked oligomers from the GDY surface, thereby exposing its intrinsic nanochannel structure. Furthermore, the Raman spectrum confirms the successful synthesis of GDY (Fig. S4). The Raman scattering peaks at 1933 and 2116 cm^−1^ are attributed to the stretching vibrations of the conjugated diyne bonds (–C≡C–C≡C–) in GDY [48]. In the annealed sample, the intensity of the vibration peaks for the conjugated diyne bonds in Fe–N-GDY decreased due to the substitution of some *sp*-hybridized C atoms by N atoms. The TEM image of Fe–N-GDY (Fig. 1e) reveals a staggered porous structure composed of stacked nanosheets. The thickness of the outer nanosheets of the microspheres is approximately 80 nm, and no Fe nanoparticles were observed. In the Double Cs-corrected TEM image of Fe–N-GDY (Fig. 1f), numerous isolated bright spots corresponding to Fe atoms are observed, indicating that Fe is dispersed in a single-atom form without aggregation. A consistent result was also observed in the Double Cs-corrected image of Fe-GDY (Fig. S5). Simultaneously, Fig. 1g presents the EDS elemental mapping of Fe–N-GDY, where C, N, and Fe elements can be seen uniformly distributed throughout the Fe–N-GDY microspheres. For comparison, a sample with high Fe loading (Fe NPs/Fe–N-GDY) was prepared. As the number of anchored Fe atoms increased, the Fe atoms began to aggregate and embed into the two-dimensional network of GDY in the form of Fe nanoparticles. The HAADF-STEM image of Fe NPs/Fe–N-GDY (Fig. S6) confirms this process. ICP-MS measurements (Table S1) revealed that the Fe elemental contents in the Fe-GDY, Fe–N-GDY, and Fe NPs/Fe–N-GDY microspheres are 1.2, 1.6, and 5.4 wt%, respectively, which aligns with the experimental design.

**Fig. 2:**
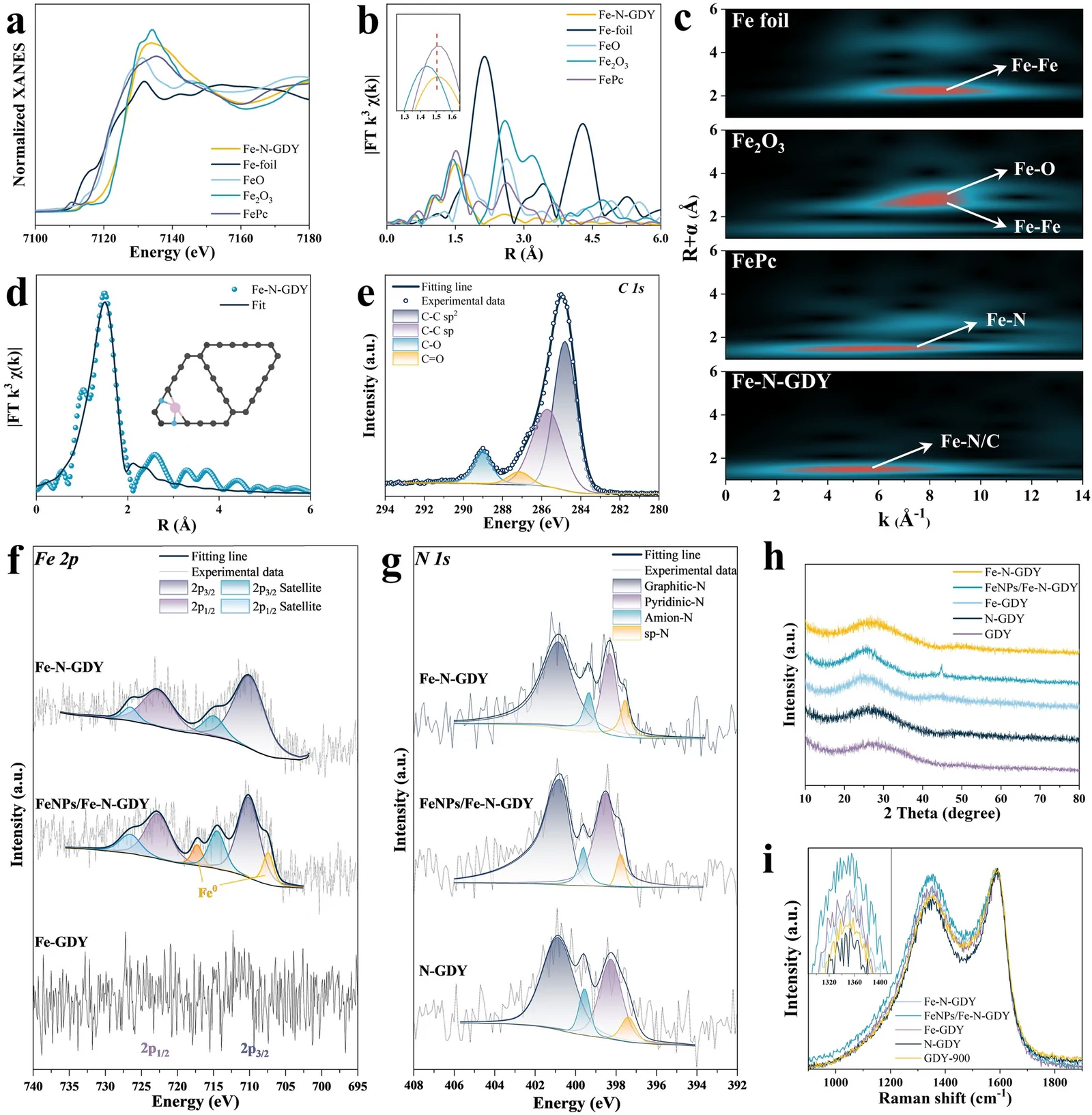
**a** Fe K-edge XANES spectra. **b** FT-EXAFS curve. **c** Wavelet transform contour plots of Fe foil, FePc, Fe_2_O_3_and Fe–N-GDY. **d** EXAFS fitting curve at d k-space. **e** C 1*s* high-resolution XPS spectrum of Fe–N-GDY. **f** Fe 2*p*, **g** N 1*s* high-resolution XPS spectrum. **h** XRD patterns and **i** Raman spectra (532 nm) of the series of samples

The chemical coordination state and electronic structure of the central Fe atom have a significant influence on the overall electrical properties of the material. To elucidate the oxidation state and coordination environment of Fe, characterization was performed using X-ray absorption fine structure (XAFS) spectroscopy. The Fe K-edge X-ray absorption near-edge structure (XANES) analysis results are presented in Fig. 2a. The absorption edge of Fe–N-GDY lies between those of FeO and Fe_2_O_3_, confirming that the charge state of the isolated Fe atoms is between + 2 and + 3. This charge characteristic originates from electron transfer between the Fe atoms and adjacent N and C atoms. In the Fourier-transformed k^3^-weighted extended X-ray absorption fine structure (EXAFS) spectra (Fig. 2b), Fe–N-GDY exhibits a main peak at 1.51 Å, positioned between those of iron phthalocyanine (FePc) and Fe_2_O_3_. Simultaneously, in the wavelet transform analysis of the EXAFS (WT-EXAFS) signal contour plot (Fig. 2c), a single intensity maximum is observed for Fe–N-GDY at 4.7 Å^−1^, its location being close to that of the FePc reference sample. Comparative analysis shows that both Fe foil and Fe_2_O_3_ reference samples exhibit distinct Fe–Fe scattering peaks at ~ 8 Å^−1^. Combining the HAADF-STEM image results with the WT-EXAFS data confirms that Fe atoms in the Fe–N-GDY system primarily exist in a single-atom form. The least-squares fitting result in R-space (Fig. 2d) indicates a coordination number for Fe close to 4. Detailed fitting structural parameters are provided in Table S2. Integrating the above analysis results, it can be concluded that the central Fe atom forms coordination bonds with two C atoms and two N atoms, embedding into the GDY framework with an FeN_2_C_2_ single-atom coordination structure. The variation of the C element in Fe–N-GDY was analyzed via its high-resolution C 1*s* XPS spectrum and its deconvoluted curves (Fig. 2e). The sub-peaks at binding energies of 284.5 and 285.3 eV correspond to carbon in *sp*^2^-hybridized and *sp*-hybridized forms [49], respectively. Their area ratio is slightly greater than the 1:2 ratio found in pristine GDY. This change stems from the transformation of some *sp*-hybridized C during the annealing process, due to defects and N atom doping [50].

To further elucidate the chemical composition and electronic structure of the materials, a series of samples were systematically analyzed using XPS, XRD, and Raman spectroscopy. Figure 2f compares the Fe 2*p* XPS spectra of three Fe-containing samples. All three samples exhibit peaks of varying intensities at 710.1 and 723.2 eV, corresponding to the typical peak positions for Fe 2*p*_3/2_ and Fe 2*p*_1/2_ [51], respectively, along with accompanying satellite peaks. This indicates that the oxidation state of Fe is predominantly Fe^2+^ and Fe^3+^. In contrast, the presence of Fe nanoclusters in Fe NPs/Fe–N-GDY results in the appearance of characteristic peaks attributable to Fe^0^ at 707.9 and 717.4 eV. The high-resolution N 1*s* XPS spectra and their deconvoluted curves for the three N-containing samples (Fig. 2g) reveal that, in addition to the typical N species—graphitic N, pyridinic N, and amino N—all exhibit a distinct *sp*–N peak at a lower binding energy (397.5 eV). The presence of this peak confirms the successful incorporation of N atoms into the graphdiyne framework. Due to differences in formation energy, N preferentially substitutes the *sp*–C atoms adjacent to benzene rings [42]. The crystalline information of the samples was analyzed by XRD. As shown in Fig. 2h, all five samples contain broad diffraction peaks belonging to GDY. Apart from the diffraction peaks attributable to Fe (ICDD/PDF No. 97-005-2258) in Fe NPs/Fe–N-GDY, no distinct diffraction peaks from crystalline iron or iron oxides were observed. This indicates excellent dispersion of Fe atoms in both Fe–N-GDY and Fe-GDY. To investigate the influence of single atoms on the defect level of GDY, the obtained samples were characterized by Raman spectroscopy. The peaks at 1346 and 1578 cm^−1^ represent the D band and G band of GDY, respectively. The D band is related to defects or edges, while the G band is associated with the C–C bond vibration mode of *sp*^2^-hybridized carbon atoms. The intensity ratio I_D_/I_G_ [52] can be used to evaluate the defect level in GDY. The Raman spectroscopy results (Fig. 2i, Table S2) show that Fe-GDY (0.9) and Fe–N-GDY (0.88) possess relatively high I_D_/I_G_ ratios. This is attributed to the introduction of Fe single atoms, which can induce defect formation and increase the disorder degree of the GDY matrix. Furthermore, the presence of a large amount of Fe leads to Fe NPs/Fe–N-GDY exhibiting the highest I_D_/I_G_ (0.95).

### Analysis of Microwave Absorption Properties of Fe–N-GDY

**Fig. 3:**
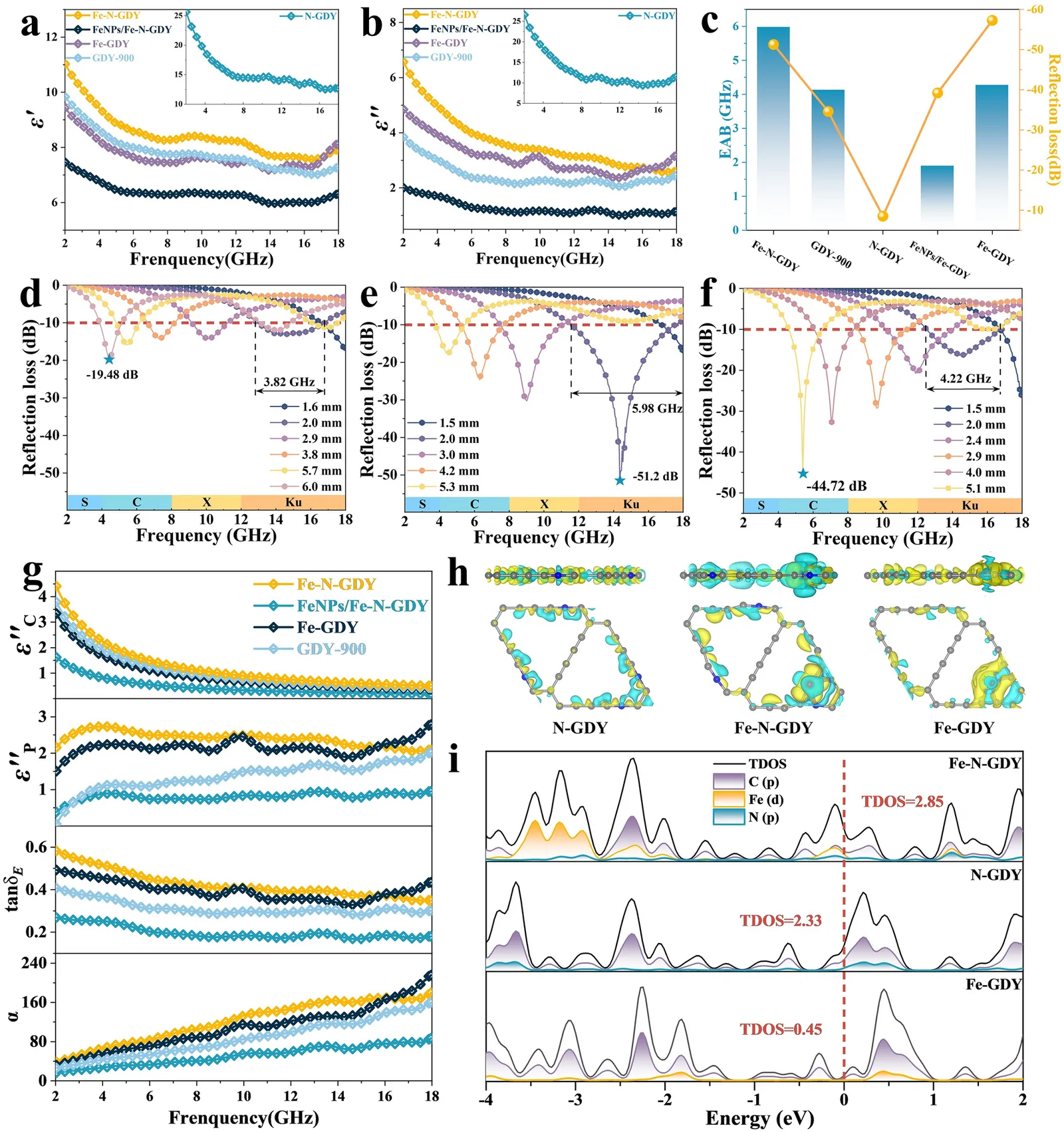
**a** Real part $\varepsilon ^{\prime }$ and **b** imaginary part $\varepsilon ^{{\prime \prime }}$ of complex permittivity. **c** Comparison of EAB and RL_min_. 2D reflection loss-frequency plots for **d** GDY-900, **e** Fe–N-GDY, and **f** Fe-GDY. **g** $\varepsilon ^{{\prime \prime }} _{C}$, $\varepsilon ^{{\prime \prime }} _{P}$, α and $\tan \delta_{E}$ of the samples. **h** Differential charge density distribution and **i** DOS of N-GDY, Fe–N-GDY and Fe-GDY

Electromagnetic parameters provide a direct reflection of a material's dielectric response behavior in an electromagnetic field. The aforementioned samples were evaluated using a vector network analyzer (VNA). The real part ($\varepsilon ^{\prime }$) and imaginary part ($\varepsilon ^{{\prime \prime }}$) of the complex permittivity for the series of absorbers, measured at a filler loading of 24 wt%, are presented in Fig. 3a, b, respectively. Within the 2–18 GHz frequency range, Fe–N-GDY exhibited a significantly enhanced dielectric response compared to GDY-900 ($\varepsilon ^{\prime }$: 9.83–7.02, $\varepsilon ^{{\prime \prime }}$: 3.81–2.05), with $\varepsilon ^{\prime }$ ranging from 11.28 to 7.59 and $\varepsilon ^{{\prime \prime }}$ from 6.57 to 2.57. In comparison, the $\varepsilon ^{\prime }$ of Fe-GDY (9.43–7.25) was lower than that of GDY-900, but it showed a higher $\varepsilon ^{{\prime \prime }}$ (4.84–2.31). This indicates that alterations in the single-atom structure have a significant influence on the dielectric response behavior of the GDY matrix. Furthermore, the Fe NPs/Fe–N-GDY sample containing iron nanoparticles showed a substantial decrease in both $\varepsilon ^{\prime }$ and $\varepsilon ^{{\prime \prime }}$ values. This is attributed to the additional contact resistance introduced by the poor interfacial contact between the nanoparticles and the carbon substrate, which severely impedes carrier migration and leads to a weakened overall conductive loss capability [53]. Among the aforementioned absorbers, N-GDY exhibited the highest $\varepsilon ^{\prime }$ and $\varepsilon ^{{\prime \prime }}$. This is because the electronegativity difference between nitrogen (3.04) and carbon (2.55) atoms drives charge redistribution. Moreover, *sp*-N hybridization effectively modulates the band structure of GDY, increasing carrier concentration and mobility [54], thereby significantly enhancing the material's conductivity [55]. The conductivity measurements of the absorber series via the four-probe method yielded results consistent with the above discussion (Fig. S7). Based on the measured electromagnetic parameters, three-dimensional reflection loss (RL) versus frequency plots for the absorber series were calculated using transmission line theory (Fig. S9). Their effective absorption bandwidth (EAB) and minimum reflection loss (RL_min_) were summarized and are presented in Fig. 3c. The performance summary clearly demonstrates that the introduction of both iron single-atom configurations (FeN_2_C_2_ and FeC_4_) effectively enhances the microwave absorption performance of GDY. Among them, Fe–N-GDY (FeN_2_C_2_) exhibited the best microwave attenuation capability, possessing both an excellent RL_min_ (−51.2 dB @ 14.3 GHz) and a broad EAB, significantly outperforming the other samples. At a matching thickness of only 2.0 mm, the EAB of Fe–N-GDY reached 5.98 GHz (12.02–18.00 GHz), representing a 57% improvement compared to GDY-900 (3.80 GHz @ 2.0 mm). In contrast, Fe-GDY with the FeC4 coordination structure (Fig. 3f) achieved a maximum EAB of 4.22 GHz (12.33–16.60 GHz) at a corresponding matching thickness of 1.9 mm. It is noteworthy that despite N-GDY having the highest conductivity and Fe NPs/Fe–N-GDY containing magnetic components, neither exhibited excellent practical microwave absorption performance.

The aforementioned results indicate that differences in the coordination environment of Fe single atoms significantly influence the dielectric properties of the materials. To clarify the structure–performance relationship between the single-atom structure and absorption capability, a more in-depth investigation into the dielectric response behavior of the samples was conducted by analyzing electromagnetic parameters and performing DFT theoretical calculations. Firstly, based on Debye theory [56], the conductive loss ($\varepsilon ^{{\prime \prime }} _{C}$) and polarization loss ($\varepsilon ^{{\prime \prime }} _{P}$) of the samples were calculated [57]. Conductive loss originates from the migration of free charge carriers under the alternating electric field, dissipating energy due to resistance, and its magnitude is often directly related to the material's electrical conductivity [19, 58]. The polarization loss mechanism involves the movement or rearrangement of internal electric dipoles within the material caused by the external electric field, which stores energy and dissipates electromagnetic waves. The results show that Fe–N-GDY exhibited the highest $\varepsilon ^{{\prime \prime }} _{C}$, while Fe-GDY, the sample without N-doping, showed a lower ($\varepsilon ^{{\prime \prime }} _{C}$) compared to GDY-900. This originates from changes in carrier concentration and the band structure of GDY induced by N-doping, which enhances the electron mobility of the absorber, optimizes electron transport pathways, and thereby endows the absorber with stronger conductive loss capability.Both Fe–N-GDY and Fe-GDY showed significantly higher ($\varepsilon ^{{\prime \prime }} _{P}$) than GDY-900, with Fe–N-GDY having the largest ($\varepsilon ^{{\prime \prime }} _{P}$). This indicates that the introduction of iron single atoms, especially the FeN_2_C_2_ configuration, greatly enhances the polarization relaxation strength of the material. This is likely due to the abundant dipoles introduced by the FeN_2_C_2_ active centers themselves and the *sp*-N-doping sites. To further quantify the loss capability of the materials, the attenuation constant (α)—frequency curves and dielectric loss tangent ($\tan \delta_{E}$)—frequency curves for the samples were calculated and plotted. The attenuation constant (α) and dielectric loss tangent ($\tan \delta_{E}$) are two key parameters for evaluating the dielectric loss capability of absorbing materials [59–61]. They represent the efficiency of converting EMW into heat and the ability to attenuate electromagnetic waves, respectively, providing a macroscopic manifestation of the material's intrinsic loss mechanisms [20, 62]. As shown in Fig. 3g, Fe–N-GDY exhibited the highest α and ($\tan \delta_{E}$) values across the entire frequency band, consistently confirming its most excellent dielectric loss capability, followed by Fe-GDY. Both were significantly superior to GDY-900. The analysis based on quarter-wavelength (λ/4) matching model showed that the experimental data points align well with the simulated λ/4 curve (Fig. S10), confirming that the microwave absorption of these MAMs follows the λ/4 cancellation mechanism. Meanwhile, Cole–Cole semicircles, representing dielectric relaxation phenomena, were observed in the $\varepsilon ^{\prime } - \varepsilon ^{{\prime \prime }}$ curves of the absorber series (Fig. S11). In addition to possessing excellent loss capability, efficient absorbers must also achieve good impedance matching with free space. To evaluate the impedance matching properties of the absorber series, two-dimensional contour maps of $\left| {Z_{in} /Z_{0} } \right|$ were plotted (Fig. S12). The yellow regions in the maps indicate the frequency range where the material achieves good impedance matching with electromagnetic waves (0.9 < $\left| {Z_{in} /Z_{0} } \right|$< 1.1). Fe–N-GDY and Fe-GDY possessed broader regions of good matching compared to GDY-900, indicating that the introduction of iron single atoms effectively optimized the impedance matching. In contrast, N-GDY and Fe NPs/Fe–N-GDY, due to their poor impedance matching characteristics, allowed only a small amount of electromagnetic wave (EMW) to enter the material interior, failing to effectively attenuate the EMW.

To elucidate the electromagnetic wave loss mechanism of single-atom-modified GDY at the electronic level, the differential charge density maps and density of states (DOS) for N-GDY, Fe-GDY, and Fe–N-GDY were obtained through DFT calculations and analysis. The differential charge density maps illustrate the redistribution of charge among atoms in the models, where isosurfaces in yellow and cyan denote regions of electron accumulation and depletion, respectively. This directional charge transfer induces the formation of local electric dipole moments. Under an alternating electric field, these dipoles undergo deformation and orientation, leading to effective attenuation of EMW. More intense charge transfer behavior typically endows the dipoles with stronger attenuation capability. The differential charge density map of N-GDY (Fig. 3h) shows that, due to the electronegativity difference between N and C, the electron cloud clearly shifted from adjacent carbon atoms toward the nitrogen atom. This not only forms a strong local dipole around the nitrogen atom but also increases the free carrier concentration in the material via an n-type doping effect, collectively enhancing its polarization response and conductive capability. In the models of Fe-GDY and Fe–N-GDY, more extensive and drastic charge redistribution was observed. This originates from: (1) the Fe atom, acting as an electron donor, introduces additional free electrons; (2) the coordinating atoms (N, C) with higher electronegativity attract electrons from the iron center. The synergistic effect of these two factors creates intense, directional charge separation around the Fe–N or Fe–C bonds, thereby constructing efficient polarization sites that contribute significantly to the dipole polarization loss of the material. In Fe–N-GDY, the Fe atom is stabilized by both *sp*-N and *sp*-C, leading to a stronger attraction for the charge around the central Fe atom. Consequently, compared to Fe-GDY with only FeC_4_ coordination, Fe–N-GDY exhibits more significant charge transfer and a stronger local polarization effect. Simultaneously, the independently existing *sp*-N in GDY can also serve as a polarization center for dipole polarization. Therefore, Fe–N-GDY contains multiple types of dipoles simultaneously induced by both the FeN_2_C_2_ units and the independent *sp*-N sites. This "multi-polarization-site synergy" mechanism is the fundamental reason for its exceptional microwave attenuation capability. To analyze the impact of single-atom doping on the electronic structure of GDY, the DOS for the corresponding models was calculated. The total density of states (TDOS) (Fig. 3i) reveals that the incorporation of both Fe and N introduces additional electrons into the system, exhibiting n-type doping characteristics. This increases the DOS near the conduction band at the Fermi level to some extent, favoring an enhancement in the carrier content within GDY. The TDOS at the Fermi level for Fe–N-GDY and N-GDY (2.33) increased compared to that of GDY (1.28), confirming that nitrogen doping can effectively enhance the metal-like conductive characteristics of GDY. The TDOS at the Fermi level for Fe–N-GDY and N-GDY (2.33) increased compared to that of GDY (1.28), confirming that nitrogen doping can effectively enhance the metal-like conductive characteristics of GDY. Since the intensity of conduction loss is directly related to the electrical conductivity of the material, the aforementioned DOS analysis results show a high degree of agreement with the experimentally measured electrical conductivity and the trend of conduction loss variation.

**Fig. 4:**
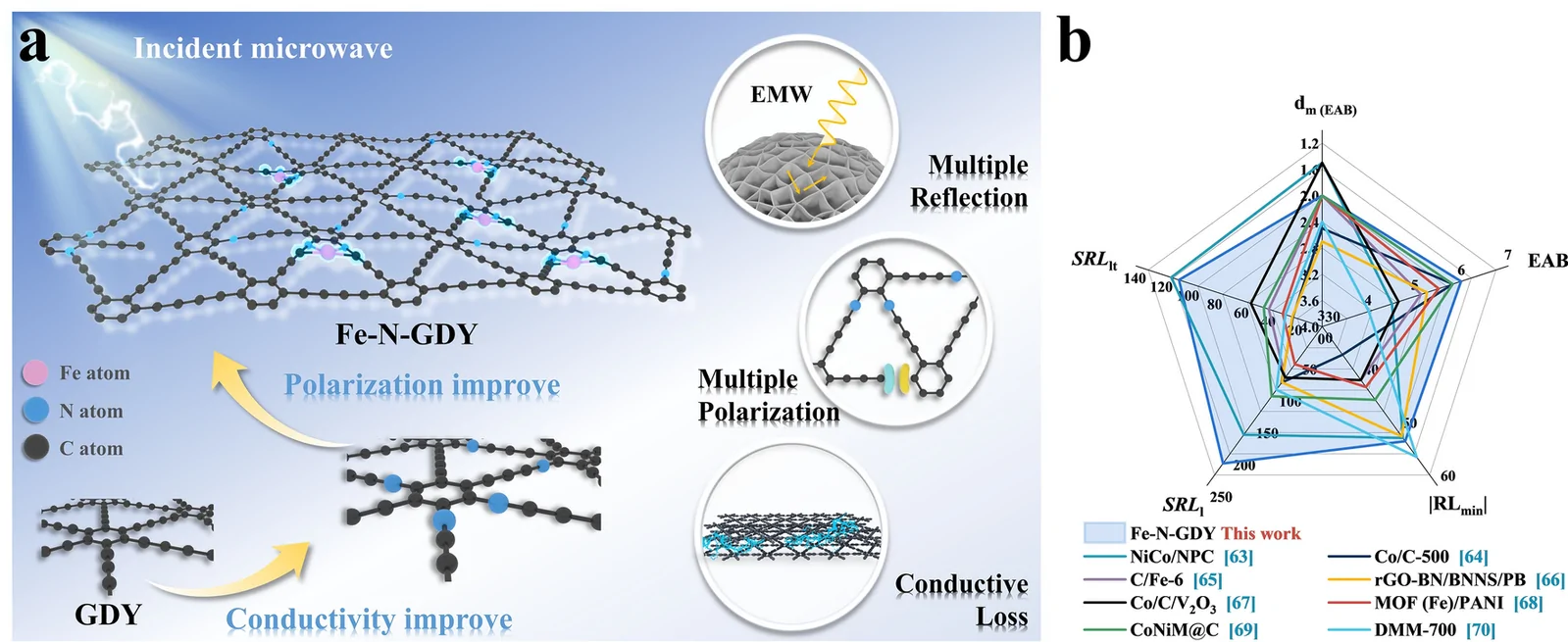
**a** Diagram illustrating the microwave absorption mechanisms of Fe–N-GDY. **b** Performance comparison between samples in this work and other metal/carbon-based composite microwave absorbers

Based on the integrated experimental characterization and theoretical calculation results, a multi-scale synergistic microwave absorption mechanism for Fe–N-GDY is proposed, as illustrated schematically in Fig. 4a. Firstly, N-doping optimizes the band structure of GDY, significantly enhancing its intrinsic conductivity, thereby strengthening conductive loss. At the atomic scale, the FeN_2_C_2_ single-atom center, formed by co-anchoring via *sp*-N and *sp*-C, acts as a highly effective polarization unit, greatly promoting dipole polarization and interfacial polarization loss. Furthermore, the excellent microwave absorption performance also stems from the synergy of multiple microwave loss mechanisms: (1) The mutually folded GDY nanosheets form a regular flower-sphere morphology, constructing a complete 3D conductive network and abundant channels. This provides more propagation paths and loss opportunities for microwaves, optimizing impedance matching. (2) The FeN_2_C_2_ single atoms, defects formed during the high-temperature annealing process, and dipoles induced by *sp*-N sites, collectively endow the absorber with multi-level dipole polarization capability. (3) The introduction of Fe and N atoms modifies the electronic properties of GDY, optimizing impedance matching and enhancing its conductive loss capacity, resulting in strong overall microwave attenuation performance of the material. To comprehensively and objectively evaluate the performance advancement of this work, a multi-index comparative analysis was conducted between the absorbers obtained in this study and previously reported ones. Radar charts were plotted based on five parameters: RL_min_, EAB, d_m(EAB)_, *SRL*_*l*_ (*SRL*_*l*_ = RL / filler loading), and *SRL*_*lt*_ (*SRL*_*lt*_ = RL / (filler loading × layer thickness)). Compared with other metal/carbon composite materials, Fe–N-GDY exhibits a larger pentagon in the radar chart. Since each dimension represents a positive performance indicator, a larger radar chart area comprehensively indicates superior overall absorption performance and more balanced metric performance of the material.

This confirms the significant comprehensive advantages of Fe–N-GDY in terms of broadband strong absorption, thin matching thickness, and lightweight characteristics.

### Microwave Absorption Properties of M–N-GDY Loaded with 3d Transition Metals

**Fig. 5:**
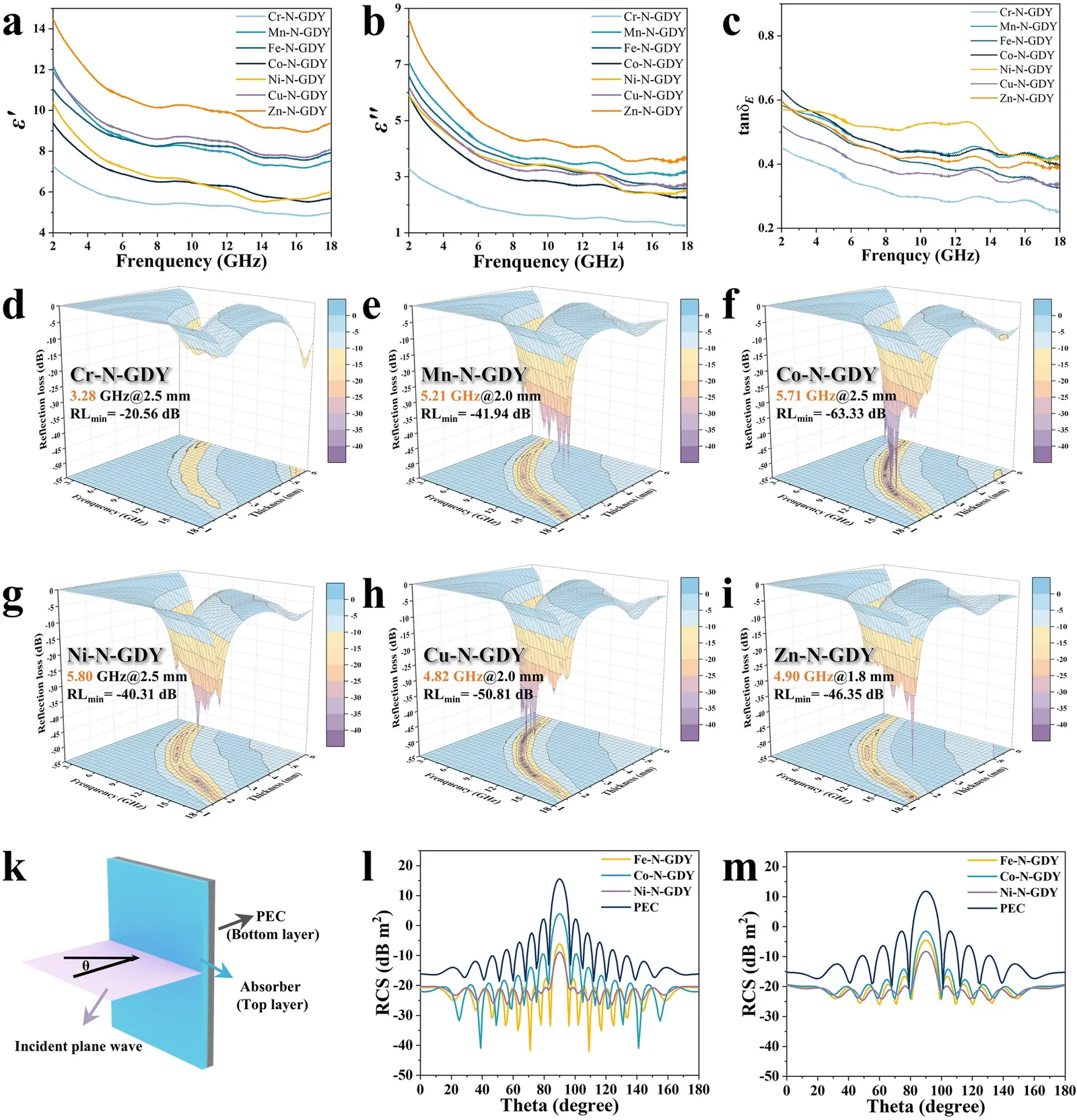
**a** $\varepsilon ^{\prime }$, **b** $\varepsilon ^{{\prime \prime }}$ and **c** $\tan \delta_{E}$ for the series of samples. 3D reflection loss-frequency diagrams: **d** Cr–N-GDY, **e** Mn-N-GDY, **f** Co–N-GDY, **g** Ni–N-GDY, **h** Cu–N-GDY, **i** Zn-N-GDY**. k** Model structure and plane wave setup in CST software. 2D RCS reflection signals for Fe–N-GDY at **l** 12 GHz and **m** 8 GHz

The aforementioned research indicates that the FeN_2_C_2_ single-atom configuration achieves excellent compatibility with the GDY substrate, effectively enhancing its microwave absorption performance. Given that different 3*d* transition metal atoms possess distinct electronic structures, different single-atom centers are expected to impart varied dielectric response characteristics to GDY. To investigate their modes of action and screen for doping metal atoms that interact favorably with GDY, six typical 3*d* single-atom M–N-GDY (M = Cr, Mn, Co, Ni, Cu, Zn) absorbers were synthesized based on the preparation system of Fe–N-GDY. Their microwave absorption performance was systematically evaluated and compared. The real part ($\varepsilon ^{\prime }$) and imaginary part ($\varepsilon ^{{\prime \prime }}$) of the complex permittivity for the seven M–N-GDY absorbers, measured at a filler loading of 24 wt%, are presented in Fig. 5a, b. The results show that differences in the central coordinating atoms lead to significant variations in the complex permittivity. Among them, Cu–N-GDY possesses the highest $\varepsilon ^{\prime }$ (14.45 – 8.94) and $\varepsilon ^{{\prime \prime }}$ (8.52 – 3.45), whereas Cr–N-GDY exhibits the lowest $\varepsilon ^{\prime }$ (7.29 – 4.82) and $\varepsilon ^{{\prime \prime }}$ (3.28 – 1.28). This substantial variation in dielectric behavior likely originates from the unique *d*-electron configurations (d^n^) and oxidation states of different 3d metal centers. These factors profoundly influence the metal-substrate electronic coupling strength, the degree of charge transfer, and consequently the magnitude of the induced dipole moments, thereby modulating their dielectric polarization and conduction behavior. The conductive loss ($\varepsilon ^{{\prime \prime }} _{C}$) and polarization loss ($\varepsilon ^{{\prime \prime }} _{P}$) for the series of materials were calculated (Fig. S13a, b) to investigate the dielectric behavior of absorbers loaded with different single atoms under an alternating electric field. For the M–N-GDY series, the ($\varepsilon ^{{\prime \prime }} _{C}$) decreases in the order: Zn > Mn > Cu > Fe > Ni > Co > Cr. Meanwhile, their ($\varepsilon ^{{\prime \prime }} _{P}$) decreases in the order: Ni > Mn > Co > Zn > Fe > Cu > Cr. Since these doping elements belong to the same period, their 3d orbital electronic structure becomes a key factor influencing the conduction and loss capabilities of the samples. This trend can be reasonably explained by the d-orbital electron filling status: When the *d*-orbitals are fully occupied by electrons, the interaction between the metal single atom and the GDY substrate is relatively weak, and the electron cloud distribution is more uniform, facilitating carrier migration and manifesting as enhanced conductive loss, as seen in Cu–N-GDY and Zn-N-GDY. In particular, the half-filled d-orbital of Cr places its electrons in a high-spin state, hindering charge carrier transfer. Consequently, Cr–N-GDY exhibits relatively low $\varepsilon ^{{\prime \prime }} _{C}$ and $\varepsilon ^{{\prime \prime }} _{P}$. Conversely, when the d-orbitals are incompletely filled, stronger d-π electron coupling exists between the metal and GDY, capable of inducing a significant local polarization field, as observed in M–N-GDY composed of Group VIII elements (Fe, Co, Ni) and Mn. The calculated $\tan \delta_{E}$ (Fig. 5c) and α (Fig. S13c) also corroborate this trend. Furthermore, 3D RL-frequency plots for the aforementioned materials were calculated based on transmission line theory (Fig. 5d–i). Overall, samples doped with Group VIII metal atoms (Fe, Co, Ni) all exhibit excellent microwave absorption (MA) performance: Fe–N-GDY achieves an EAB of 5.98 GHz@2.0 mm; Co–N-GDY has an EAB of 5.71 GHz@2.5 mm; Ni–N-GDY’s EAB covers 5.80 GHz@2.5 mm. In comparison, the EAB values for the other samples are: Mn-N-GDY (5.21 GHz@2.0 mm), Zn-N-GDY (4.90 GHz@1.8 mm), Cu–N-GDY (4.82 GHz@2.0mm), and Cr–N-GDY (3.28 GHz@2.5 mm). It can be concluded that the incompletely filled d-orbitals with a higher number of electrons in Group VIII metals are most conducive to achieving a balance between strong polarization loss and moderate conductive loss in GDY, while synergistically optimizing impedance matching (Fig. S14). Analysis of the Cole–Cole semicircles for the above samples (Figs. S15 and S11b) shows that Group VIII elements (Fe, Co, Ni) exhibit larger semicircles in the Ku band, indicating stronger dielectric relaxation processes, which correspond to their excellent broadband absorption performance.

To evaluate the application potential of these excellent absorbers in practical radar stealth scenarios, radar cross section (RCS) simulations were performed using CST software for three M–N-GDY (M=Fe, Co, Ni) samples with superior MA performance from the above series, aiming to investigate the electromagnetic wave absorption capability of the materials under realistic application conditions. The simulation model setup is shown in Fig. 5k: The material was coated onto a perfect electric conductor (PEC) plate, and the monostatic RCS varying with the incident angle was calculated at three typical radar frequencies of 4, 8, and 12 GHz. The RCS signal is a core measure of a target's scattering ability to radar waves, directly related to the difficulty of the target being detected by radar. A reduction in RCS signal strength indicates that the MAMs exhibit more beneficial radar stealth effects, with a larger RCS reduction representing stronger capability. By comparing the 3D RCS reflection signals of each sample against the PEC (Fig. S16), it was found that all three samples exhibited certain RCS attenuation capabilities at 4, 8, and 12 GHz. In comparison, Fe–N-GDY demonstrated the strongest attenuation capability for EMW at 12 GHz (Fig. 5l), with a maximum RCS reduction reaching 38.74 dB m^2^ at an incident angle of 110°. Meanwhile, Ni–N-GDY showed the strongest attenuation capability for EMW at 8 GHz (Fig. 5m), with a maximum reduction of 20.17 dB m^2^ at a corresponding incident angle of 90°. This indicates that the M–N-GDY single-atom materials developed in this work possess excellent practical radar wave attenuation capability.

## Conclusion

This study successfully developed a novel strategy to enhance the microwave absorption performance of graphdiyne by precisely tuning the coordination microenvironment of 3d transition metal single atoms. Using flower-ball-like GDY particles as the substrate, the precise regulatory effects of single-atom coordination structures (FeN_2_C_2_ and FeC_4_) and the central metal atom on the electronic structure of GDY were investigated, along with their mechanism of action in modulating the conduction and polarization properties of the absorbers. The research demonstrates that the unique sp/sp^2^-hybridized two-dimensional network of GDY provides an ideal electronic platform for the introduction of single atoms. Fe single atoms co-anchored by *sp*-N and *sp*-C bonds enhance the polarization capability of the GDY-based absorber, optimize impedance matching, and achieve efficient microwave absorption. Further investigation revealed that M–N-GDY exhibits excellent compatibility with Group VIII elements (Fe, Co, Ni), which is significantly superior to other 3d transition metal single atoms. The optimal sample, Fe–N-GDY, achieved an EAB of 5.98 GHz at a low thickness of 2.0 mm, with an RL_min_ reaching −51.2 dB. Mechanistic studies revealed that the performance enhancement of GDY originates from the synergy of multiple mechanisms: metal single atoms, *sp*-N sites, and defect sites in GDY collectively induce multiple dipole polarizations; the introduction of Fe and N atoms modulates the electrical properties of GDY and optimizes impedance matching; the three-dimensional porous conductive network induces multiple scattering, prolonging the microwave propagation path. This work pioneers the introduction of single-atom doping engineering into the GDY-based microwave absorbing material system, providing theoretical support and a technical paradigm for the design of novel electromagnetic protection materials.

## Supplementary Information

Below is the link to the electronic supplementary material.

Supplementary file1 (DOCX 4844 kb)
