# Supplementary material for: Synergistic sp-C/sp-N Anchoring of Metal Single Atoms on Graphdiyne for Enhanced Microwave Absorption
Source: Nanomicro Lett. 2026 Apr 28;18:345. doi: 10.1007/s40820-026-02187-8 (PMC13125446; doi:10.1007/s40820-026-02187-8)
Supplement: Supplementary file 1 — Supplementary file1 (DOCX 4844 kb) [file 40820_2026_2187_MOESM1_ESM.docx]

Supporting Information for

**Synergistic *sp*-C/*sp*-N Anchoring of Metal Single Atoms on Graphdiyne for Enhanced Microwave Absorption**

Yihao Fan ^1, 2^, Haichuan Cheng ^1^, Pengyu Deng ^1^, Jianfeng Wu ^1, 2,※^ , Baoliang Zhang ^1, 3, ※^

^1^ School of Chemistry and Chemical Engineering, Northwestern Polytechnical University, Xi’an710072, People’s Republic of China

^2^ Xi’an Key Laboratory of Functional Organic Porous Materials, Northwestern Polytechnical University, Xi’an 710129, People’s Republic of China

^3^ Shaanxi Engineering and Research Center for Functional Polymers on Adsorption and Separation, Sunresins New Materials Co. Ltd., Xi’an 710072, People’s Republic of China

※Corresponding authors. E-mail: [jfwu@nwpu.edu.cn](mailto:jfwu@nwpu.edu.cn) (Jianfeng Wu); [blzhang@nwpu.edu.cn](mailto:blzhang@nwpu.edu.cn) (Baoliang Zhang)

**S1 Experimental Section**

**S1.1 Microwave absorption test**

The electromagnetic parameters of the powder samples were measured by a vector network analyzer (ZNLE18, Rohde & Schwarz) using the coaxial method. The samples were mixed with paraffin wax, and then coaxial rings with an inner diameter of 3.04 mm and an outer diameter of 7 mm were machined with a mold. The filler content of all the samples was 24%.

Reflection loss (RL) is an important evaluation index of electromagnetic wave absorption performance. According to the transmission line theory, RL can be calculated by Eqs. S1 and S2:

|  | $Z=\left\vert\frac{Z_{in}}{Z_{O}} \right\vert=\sqrt{\left\vert{\mu_{r}}/{\varepsilon_{r}} \right\vert} tanh\left[ j\left( {2\pi fd}/c \right)\sqrt{\mu_{r}\varepsilon_{r}} \right]$ | (S1) |
| --- | --- | --- |

|  | $\mathrm{RL}\left( \mathrm{dB} \right)=20 \log_{10}\left\vert\frac{Z_{\mathrm{in}}-Z_{0}}{Z_{\mathrm{in}}+Z_{0}} \right\vert$ | (S2) |
| --- | --- | --- |

According to Debye's theory, the imaginary part of the complex permittivity ($\varepsilon^{''}$) can be divided into conduction loss ($\varepsilon_{C}^{''}$) and polarisation loss ($\varepsilon_{P}^{''}$) by means of Eqs. S3 and S4:

|  | $\varepsilon^{''}=\frac{\omega\tau(\varepsilon_{s}-\varepsilon_{\infty})}{1+\omega^{2}\tau^{2}}+\frac{\sigma}{\omega\varepsilon_{0}}=\varepsilon_{p}^{''}+\varepsilon_{C}^{''}$ | (S3) |
| --- | --- | --- |
|  | $\varepsilon_{C}^{''}=\frac{\alpha}{\omega\varepsilon_{0}}$ | (S4) |

The attenuation constant ($\alpha$) was calculated by Eqs. S5:

|  | $\alpha=\sqrt{\left( \mu^{''}\varepsilon^{''}-\varepsilon^{'}\mu^{'} \right)+\sqrt{{(\mu^{''}\varepsilon^{''}-\varepsilon^{'}\mu^{'})}^{2}+{(\mu^{''}\varepsilon^{''}+\varepsilon^{'}\mu^{'})}^{2}}}$ | (S5) |
| --- | --- | --- |

**S1.2 CST simulation**

Radar cross section (RCS) was simulated using CST Studio Suite 2022. Four models were simulated. In the radar cross-section simulation, the model consists of an upper absorbing layer and a lower perfect electrical conductor (PEC) layer with thicknesses of 2.0 mm and 1.0 mm, respectively. In addition, the square model was designed with a side length of 200 mm. A linearly polarized plane wave was defined as the excitation port, with the microwave propagating in the negative direction along the Y-axis and the direction of the magnetic field along the Z-axis. Open boundary conditions were used in this direction. The radar RCS of the simulated sample was represented by the following Eq. S6:

|  | $\sigma(dBm^{2} )=10\log\left[ {\frac{4\pi S}{\lambda^{2}}\left\vert\frac{E_{s}}{E_{i}} \right\vert}^{2} \right]$ | (S6) |
| --- | --- | --- |

Where $S$ is the area of the simulated plate, $\lambda$ is the length of the incident microwave, Es is the electric field strength of the transmitted wave, and $Ei$ is the electric field strength of the received wave.

**S1.3 DFT calculation**

All the DFT calculations were conducted based on the Vienna Ab initio Simulation Package (VASP). The exchange-correlation potential was described by the Perdew−Burke−Ernzerhof (PBE) generalized gradient approach (GGA). The electron-ion interactions were accounted by the projector augmented wave (PAW). All DFT calculations were performed with a cut-off energy of 400 eV, and the 3×3×1 Gamma centered Monkhorst-Pack grids k-points were selected to sample the Brillouin zone integration. The energy and force convergence criteria of the self-consistent iteration were set to 10-4 eV and 0.02 eV Å-1, respectively. DFT-D3 method was used to describe van der Waals (vdW) interactions.

**S2 Supplementary Figures and Tables**


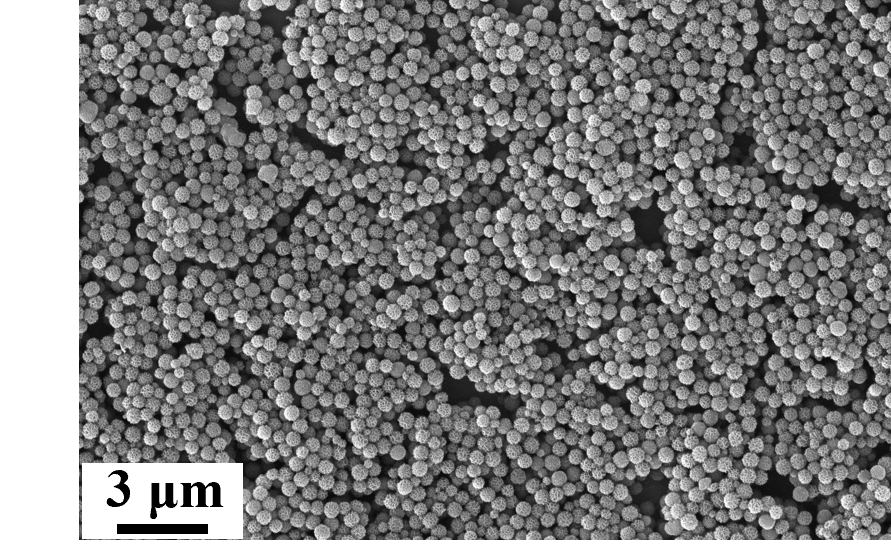


**Fig. S1** SEM figure of Cu_2_O/GDY

**
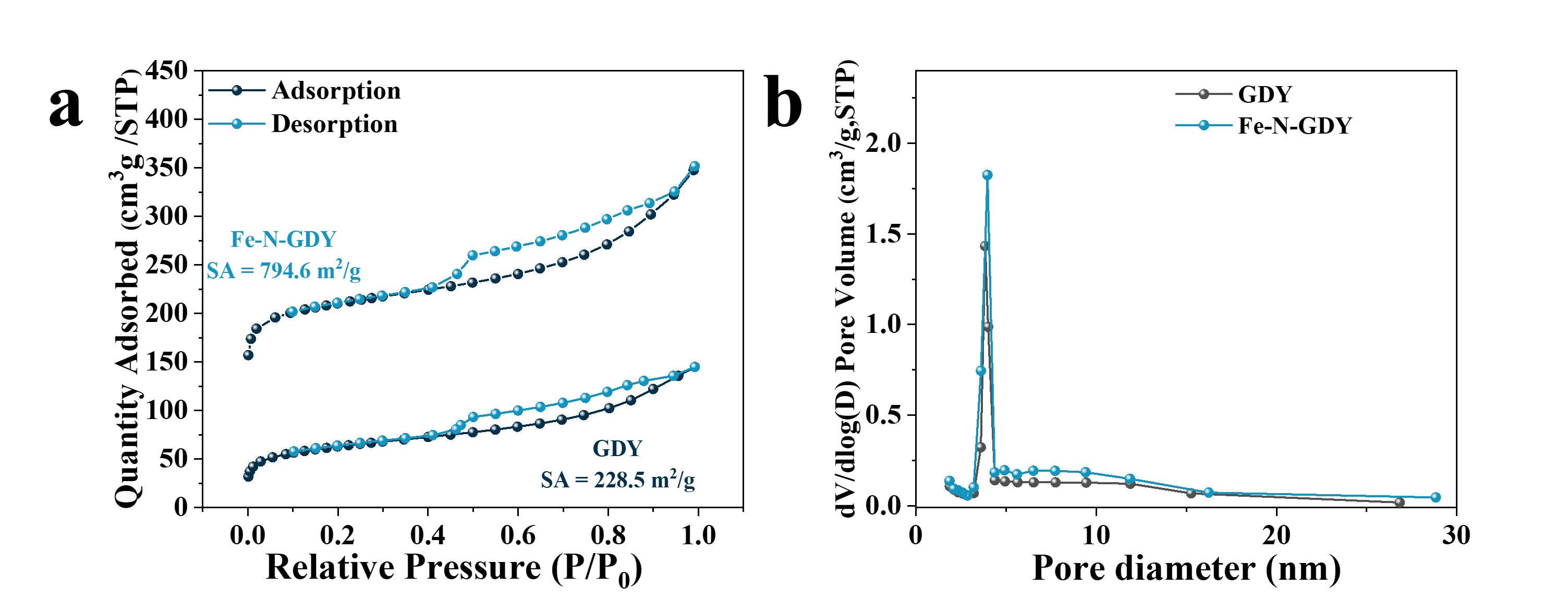
**

**Fig. S2 a** Nitrogen adsorption-desorption isotherms and **b** pore size distribution curves


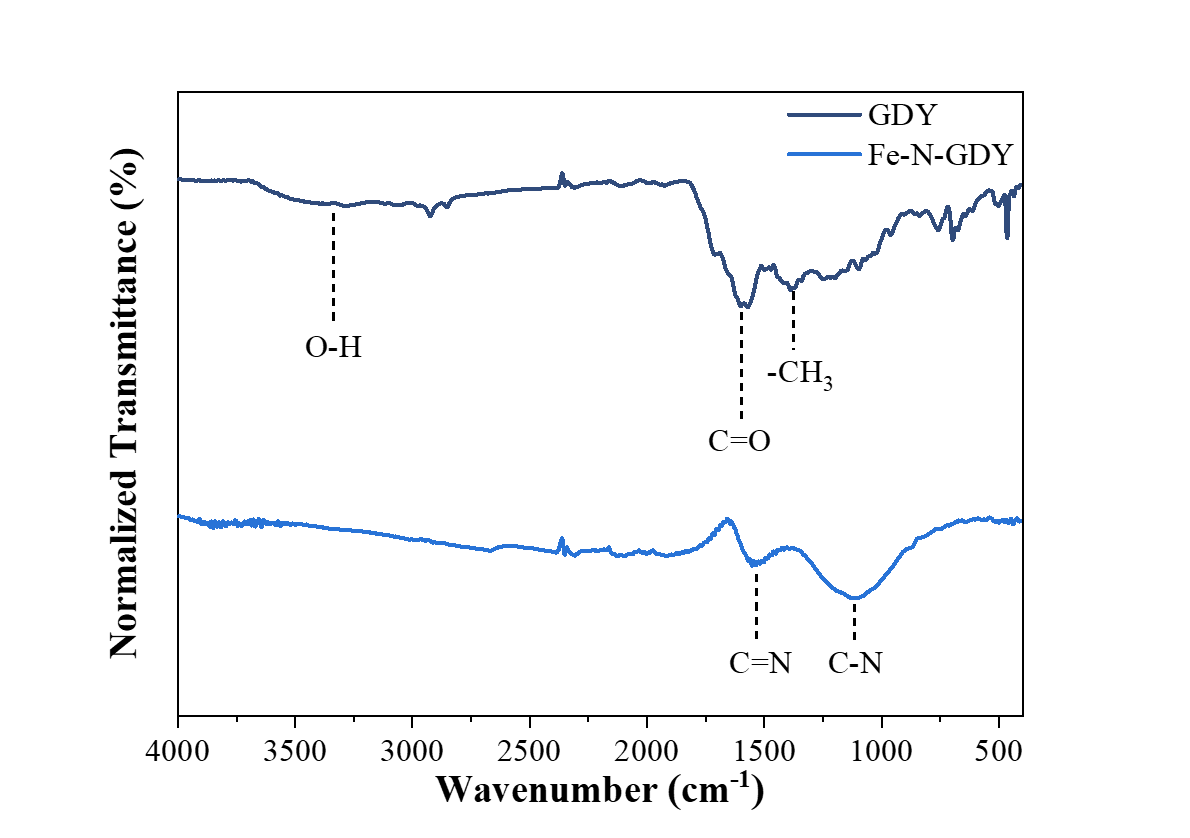


**Fig. S3** FTIR curves of GDY and Fe-N-GDY


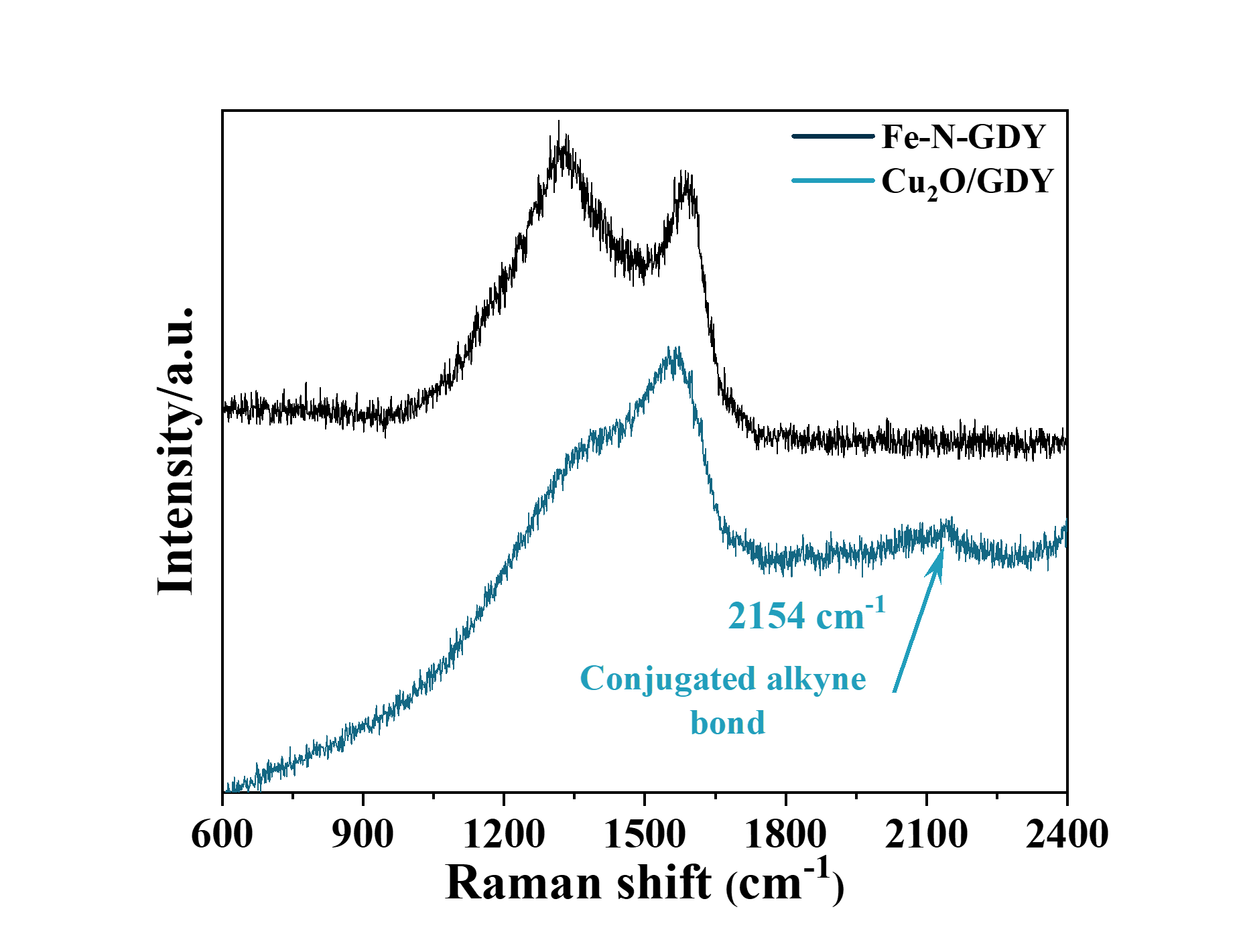


**Fig. S4** Raman spectra of Cu_2_O/GDY


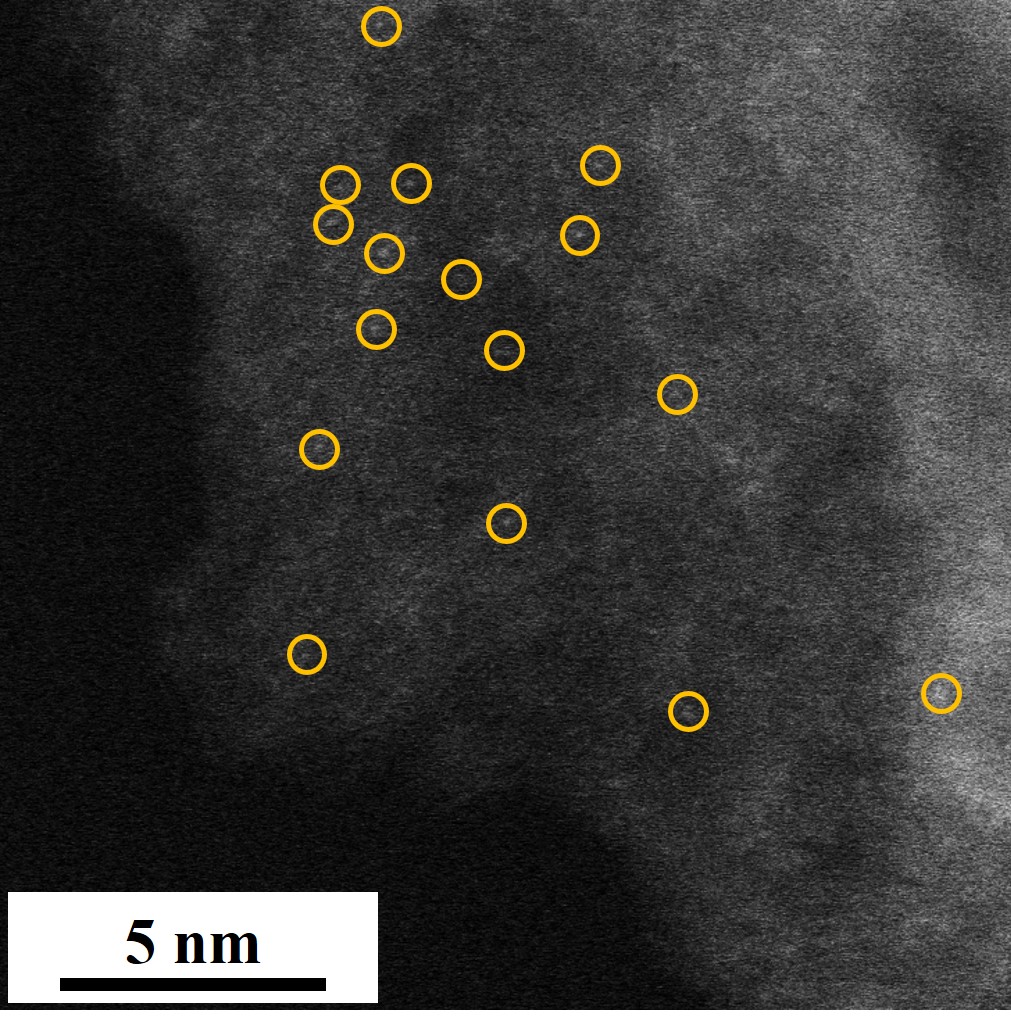


**Fig. S5** Double Cs-corrected image of Fe-GDY


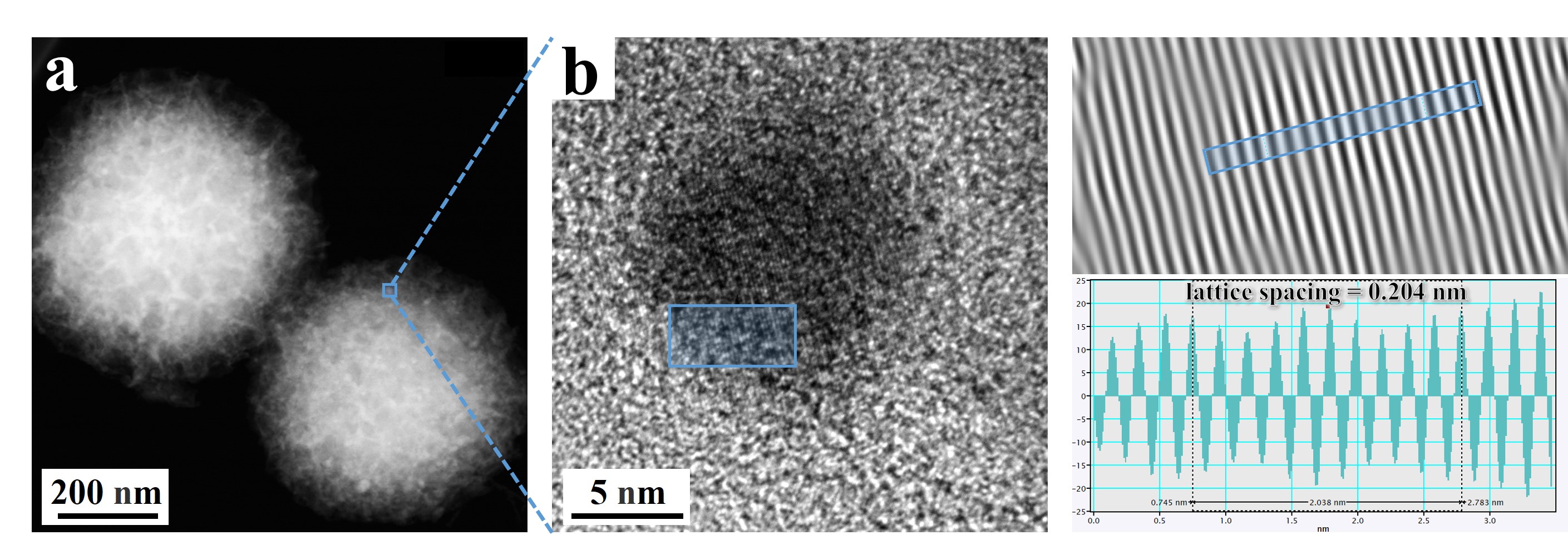


**Fig. S6 a** HAADF-STEM image and **b** lattice analysis of Fe NPs/Fe-N-GDY


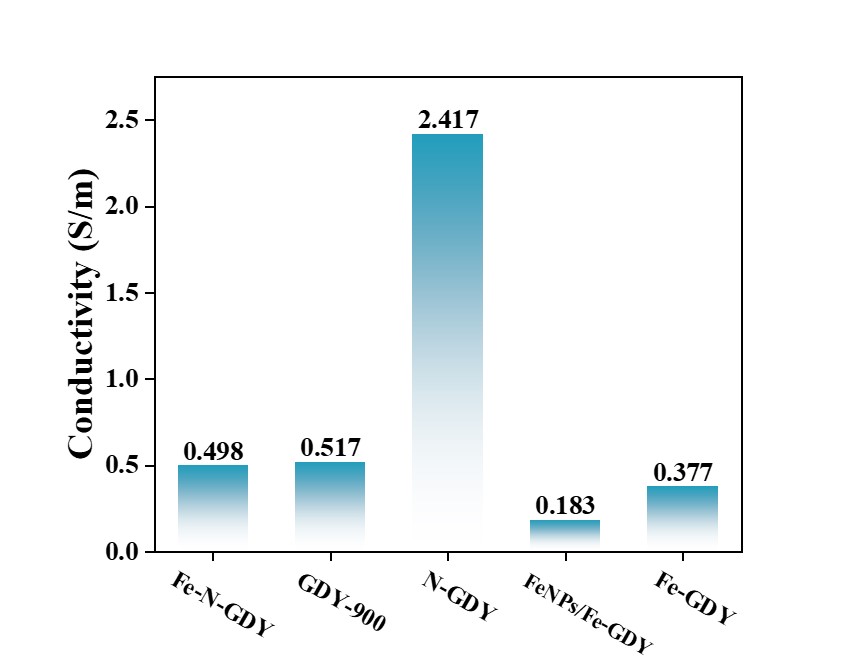


**Fig. S7** Electrical conductivity measurement via the four-probe method


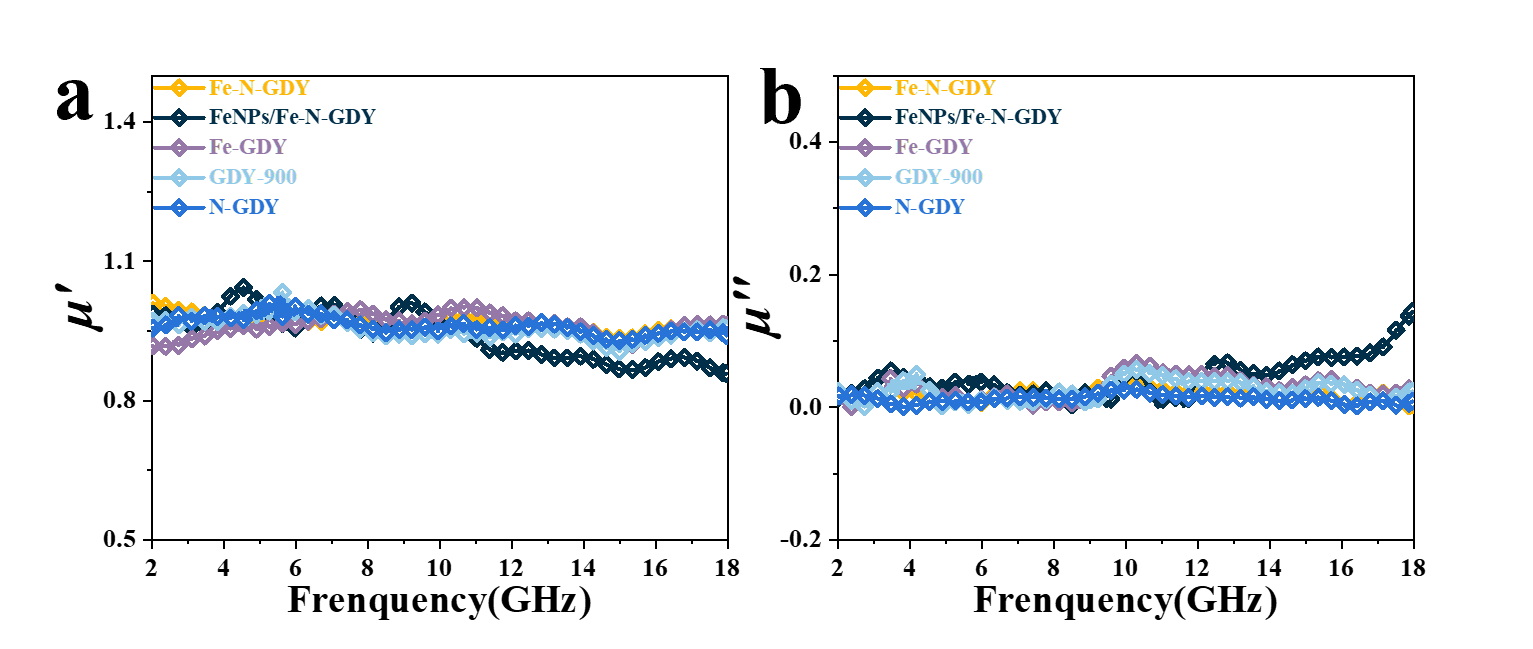


**Fig. S8 a** Real part $\varepsilon^{'}$ and **b** imaginary part $\varepsilon''$ of complex permittivity


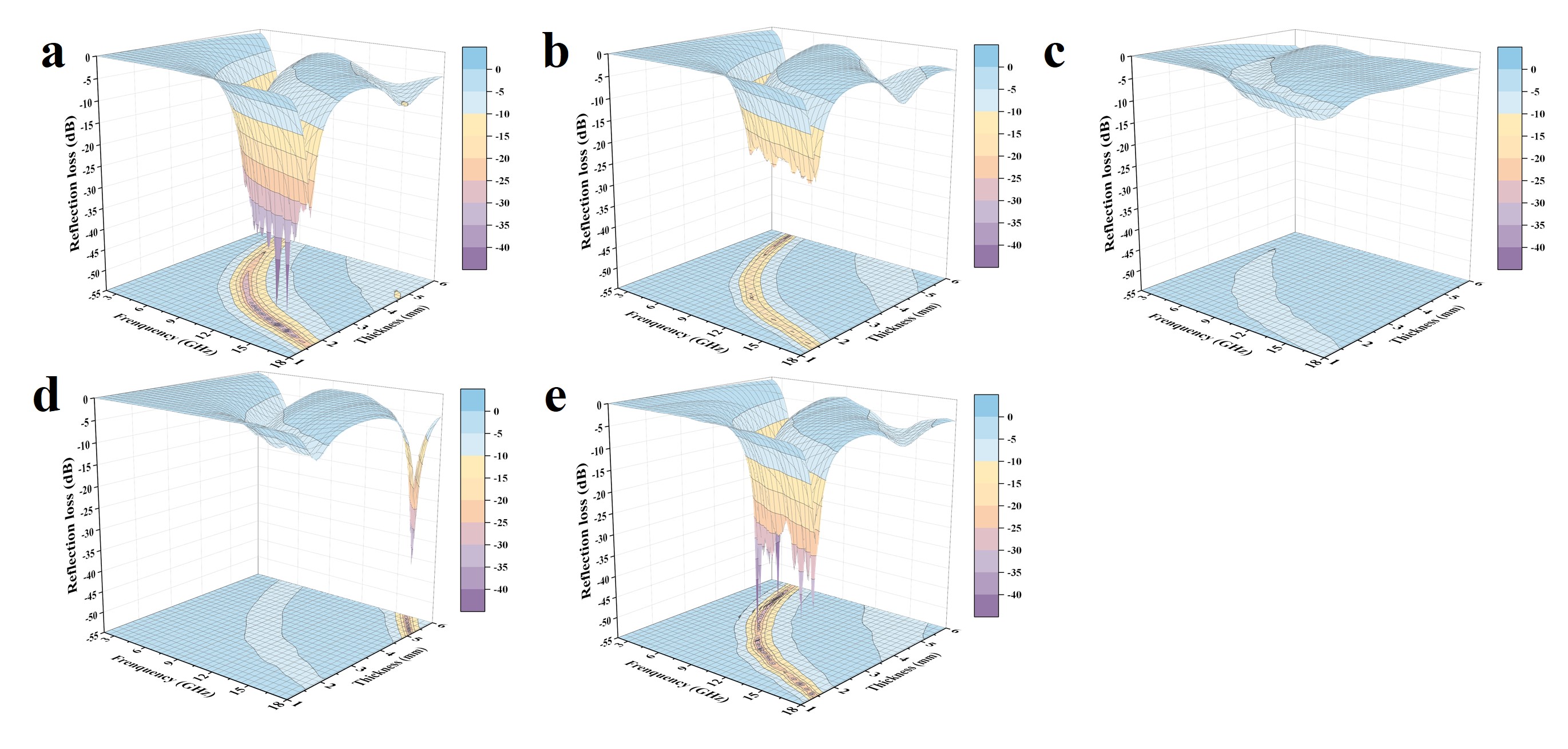


**Fig. S9** 3D RL-frequency plots of the series of microwave absorbers: **a** Fe-N-GDY, **b** GDY-900, **c** N-GDY, **d** Fe NPs/Fe-N-GDY, **e** Fe-GDY


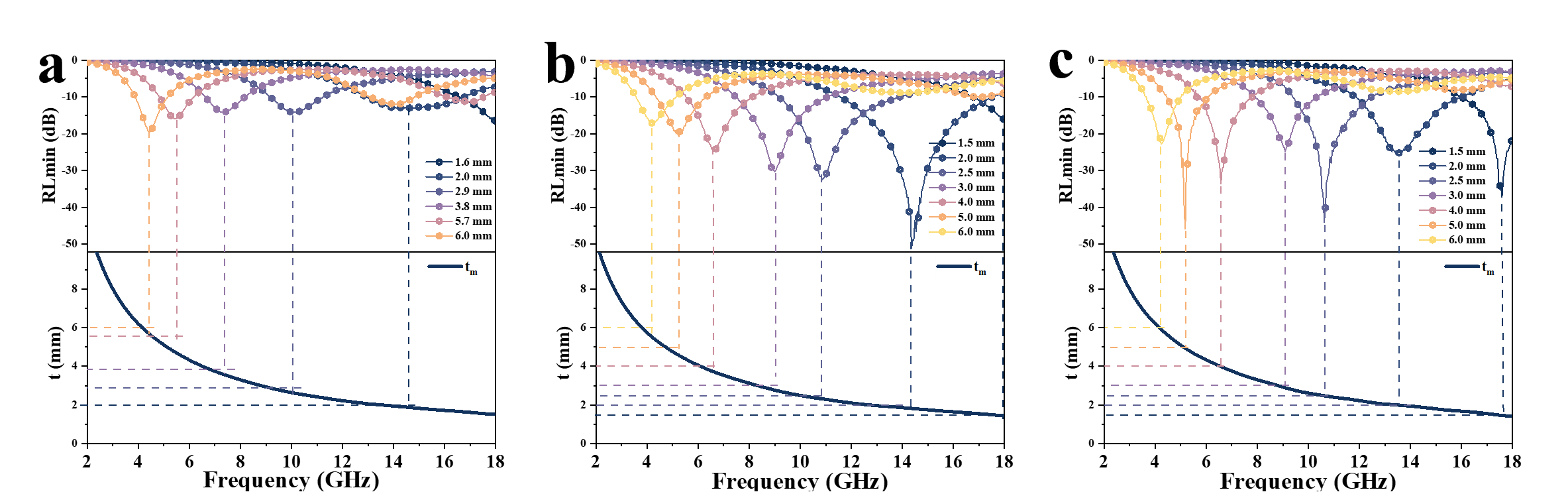


**Fig. S10** Plots of reflection losses and λ/4 with frequency of **a** GDY-900, **b** Fe-N-GDY and **c** Fe-G


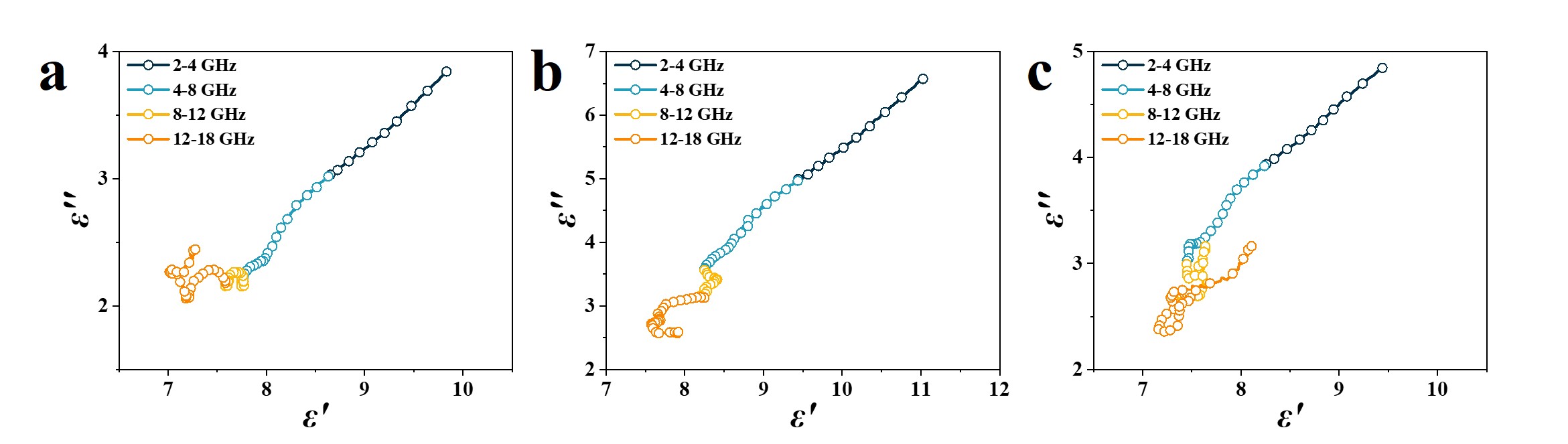


**Fig. S11** $\varepsilon^{'}-\varepsilon''$ curves of **a** GDY-900, **b** Fe-N-GDY and **c** Fe-GDY


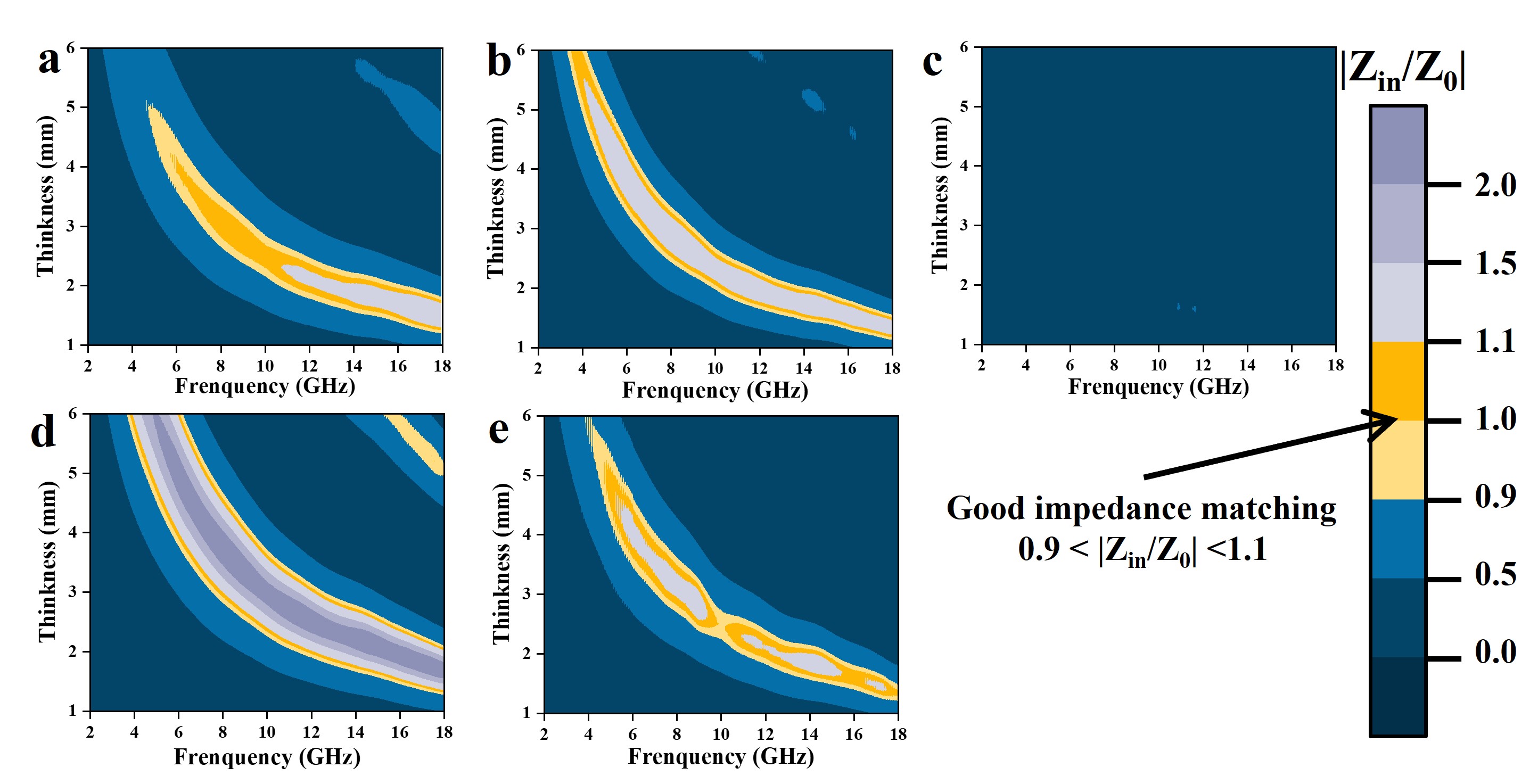


**Fig. S12** |Z_in_/Z_0_| two-dimensional contour maps of **a** Fe-N-GDY, **b** GDY-900, **c** N-GDY, **d** Fe NPs/Fe-N-GDY, **e** Fe-GDY


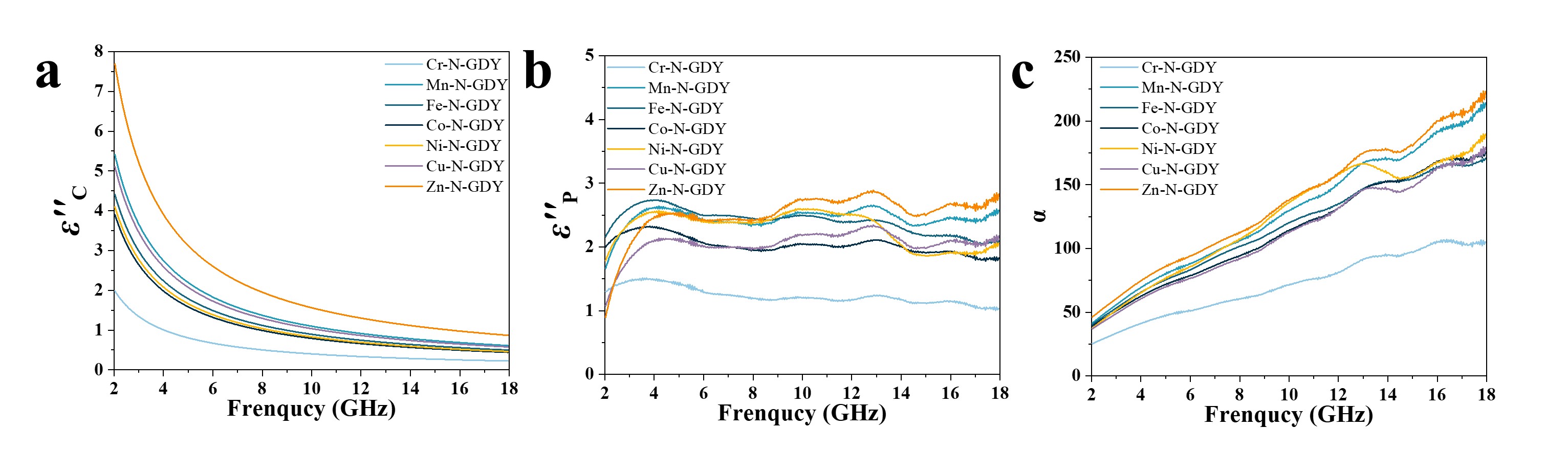


**Fig. S13** **a** ${\varepsilon''}_{C}$, **b** ${\varepsilon''}_{P}$, and **c** $ɑ$ of the samples


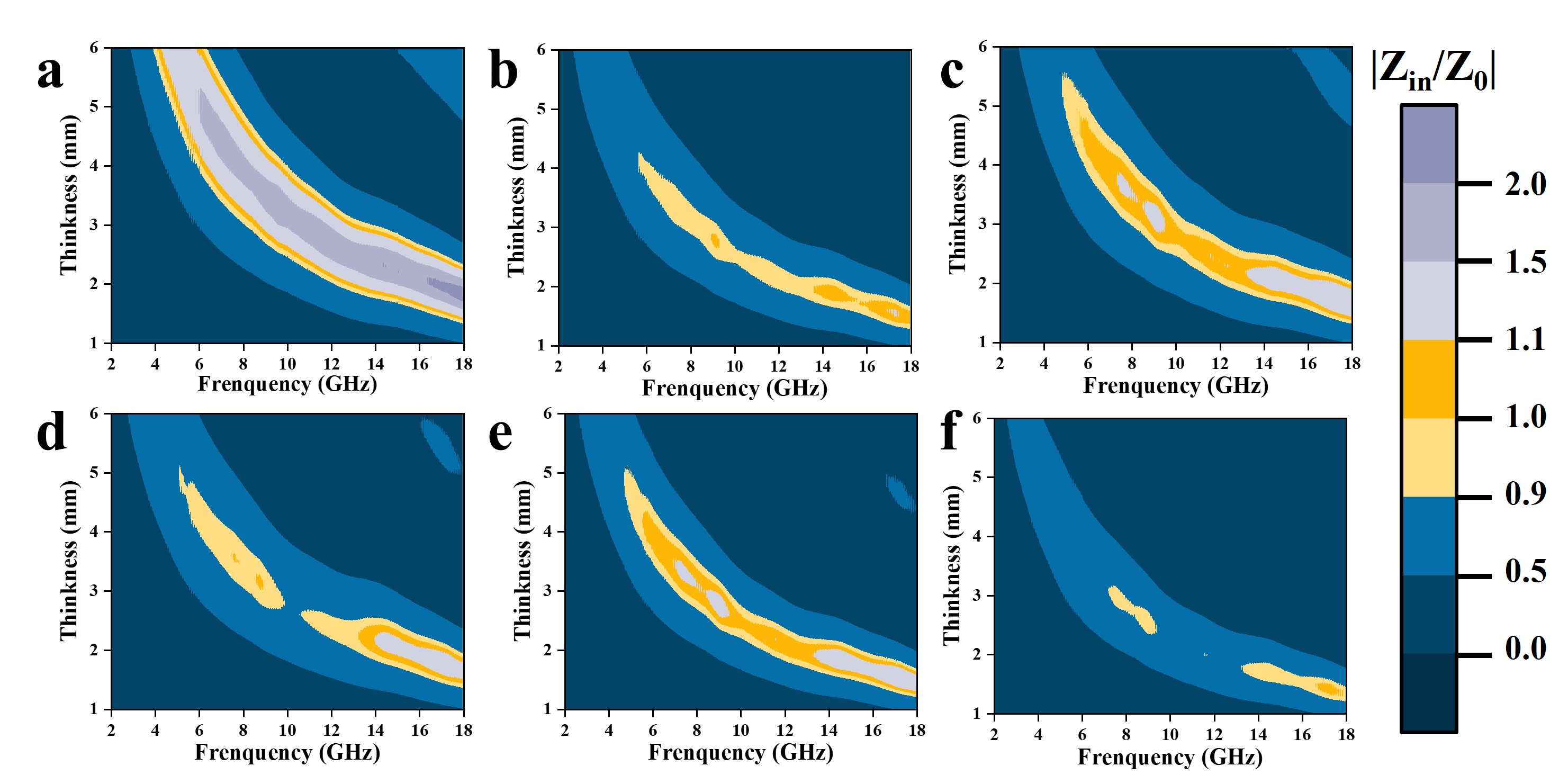


**Fig. S14** |Z_in_/Z_0_| two-dimensional contour maps of **a** Cr-N-GDY, **b** Mn-N-GDY, **c** Co-N-GDY, **d** Ni-N-GDY, **e** Cu-N-GDY, and **f** Zn-N-GDY


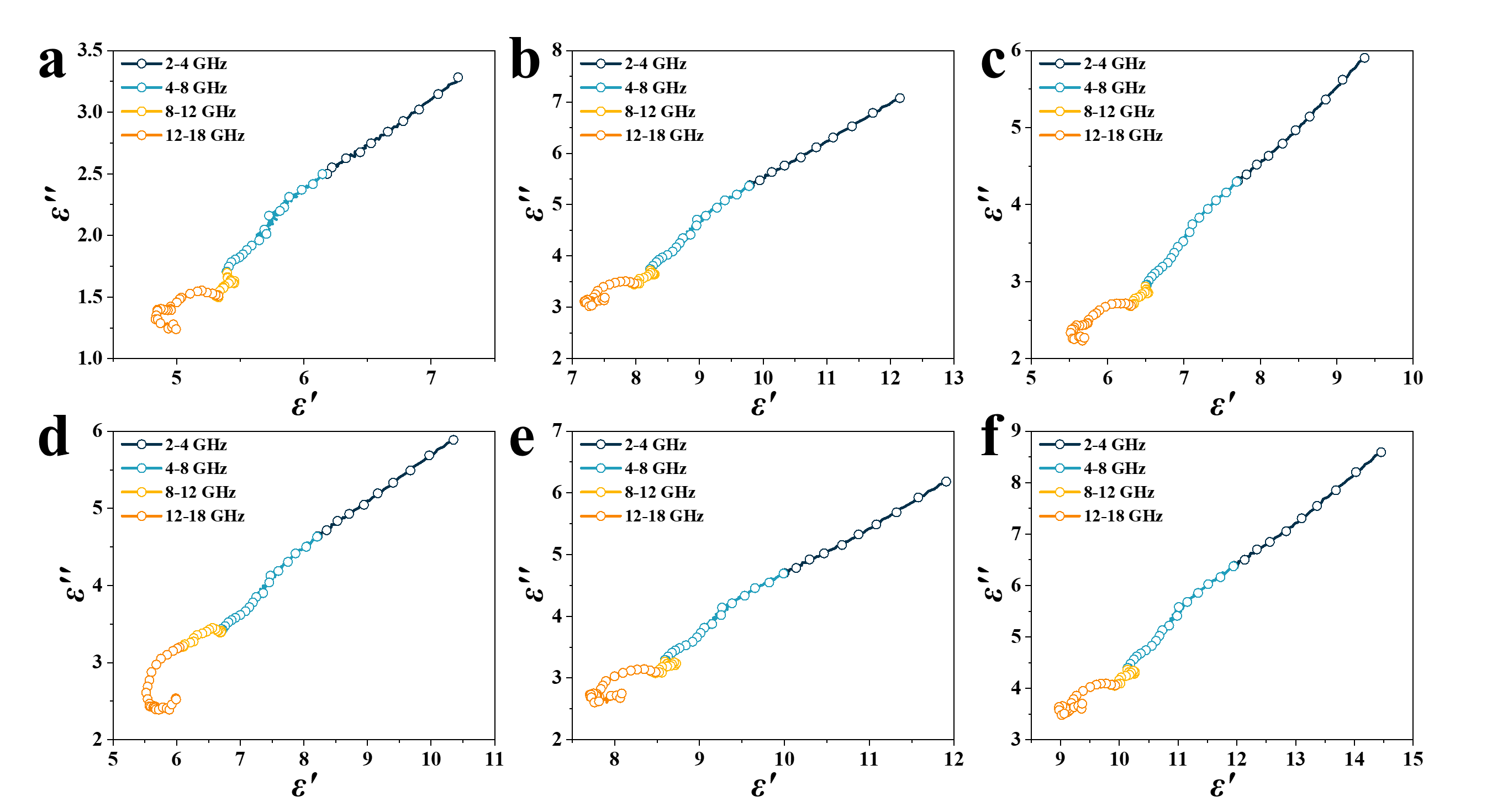


**Fig. S15** Cole-Cole plots of **a** Cr-N-GDY, **b** Mn-N-GDY, **c** Co-N-GDY, **d** Ni-N-GDY, **e** Cu-N-GDY, and **f** Zn-N-GDY


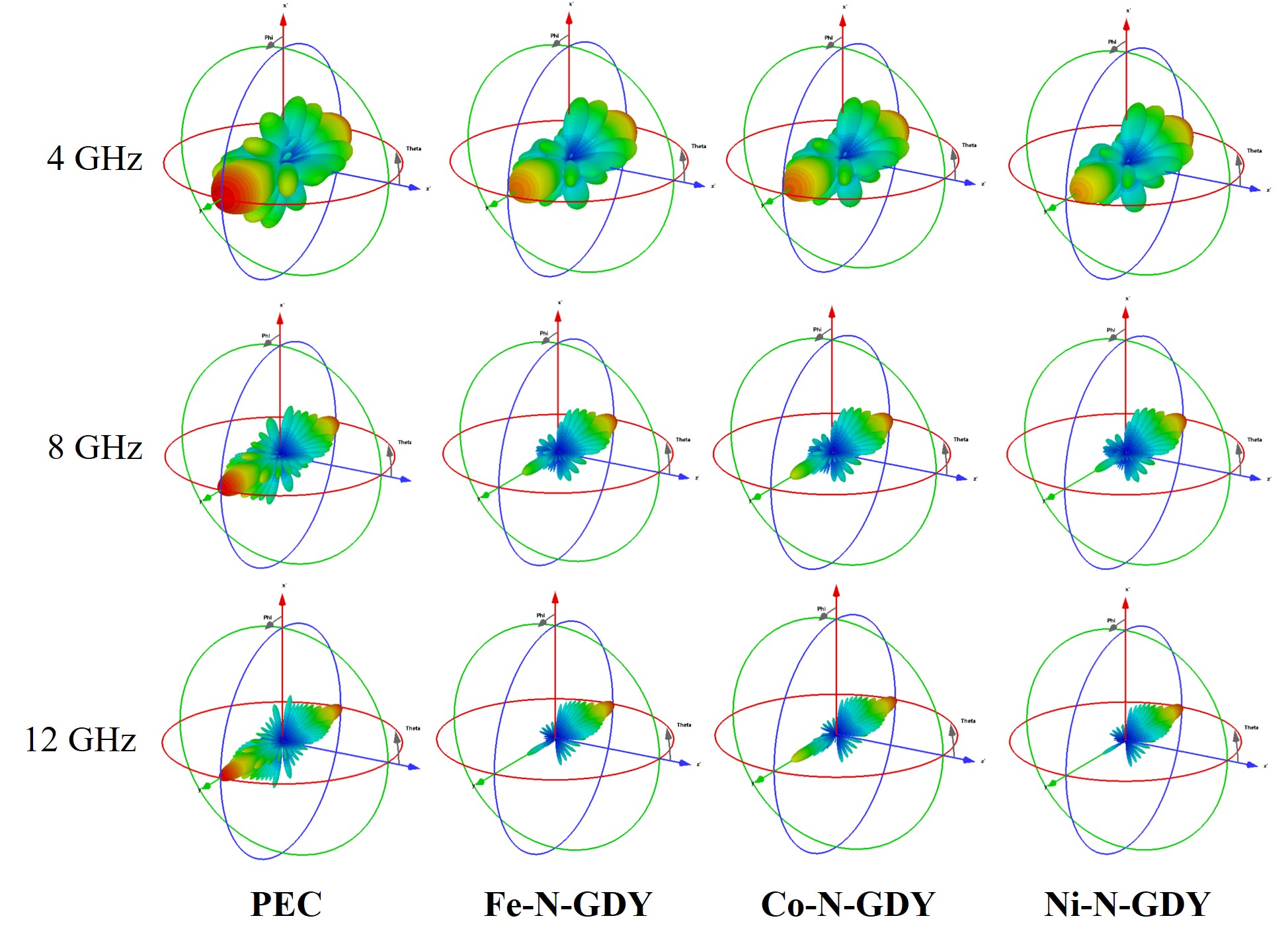


**Fig. S16** 3D RCS reflection signals of each sample at 4 GHz, 8GHz and 12GHz

**Table S1** The elemental contents determined by ICP-MS

| **Samples** | **Fe-GDY** | **Fe-N-GDY** | **Fe NPs/Fe-N-GDY** |
| --- | --- | --- | --- |
| Fe wt% | 1.2 | 1.6 | 5.4 |

**Table S2** Curvefit parameters for Fe K-edge EXAFS for Fe-N-GDY

| **Path** | **C.N.** | **R (Å)** | **σ^2^ (Å^2^)** | **ΔE_0_ (eV)** | **R factor** |
| --- | --- | --- | --- | --- | --- |
| Fe-N | 2 | 1.92±0.04 | 0.012±0.003 | -6.0±2.6 | 0.019 |
| Fe-C | 2 | 2.03±0.04 |  |  |  |

C. N. is the coordination number; R is interatomic distance; σ^2^ is Debye-Waller factor (the Mean Square Relative Displacement (MSRD)); ΔE_0_ is inner potential correction. R factor is used to value the goodness of the fitting. Data ranges:2.5≤k≤14 Å,1.0≤R≤3.0 Å. The number of variables is 4.

**Table S3** I_D_/I_G_ of the related samples

| **Samples** | **Fe-N-GDY** | **Fe NPs/Fe-N-GDY** | **Fe-GDY** | **N-GDY** | **GDY-900** |
| --- | --- | --- | --- | --- | --- |
| I_D_/I_G_ | 0.88 | 0.95 | 0.90 | 0.81 | 0.84 |
